# Genetic structure and diversity of *Nodularia douglasiae* (Bivalvia: Unionida) from the middle and lower Yangtze River drainage

**DOI:** 10.1371/journal.pone.0189737

**Published:** 2017-12-20

**Authors:** Xiongjun Liu, Yanling Cao, Taotao Xue, Ruiwen Wu, Yu Zhou, Chunhua Zhou, David T. Zanatta, Shan Ouyang, Xiaoping Wu

**Affiliations:** 1 Poyang Lake Key Laboratory of Environment and Resource Utilization (Nanchang University), Ministry of Education, Nanchang, People’s Republic of China; 2 School of Resource, Environment and Chemical Engineering, Nanchang University, Nanchang, People’s Republic of China; 3 School of Life Sciences, Nanchang University, Nanchang, People’s Republic of China; 4 Central Michigan University, Institute for Great Lakes Research, Biology Department, Biosciences 2408, Mount Pleasant, MI, United States of America; Glasgow Caledonian University, UNITED KINGDOM

## Abstract

The Yangtze River drainage in China is among the most species rich rivers for freshwater mussels (order Unionida) on Earth with at least 68 species known. The freshwater mussels of the Yangtze River face a variety of threats with indications that species are declining in abundance and area of occupancy. This study represents the first analyses of the genetic structure and diversity for the common and widespread freshwater mussel *Nodularia douglasiae* based on microsatellite DNA genotypes and mitochondrial DNA sequences. Phylogenetic analysis a fragment of the COI mitochondrial gene indicated that *N*. *douglasiae* collected from across the middle and lower Yangtze River drainage are monophyletic with *N*. *douglasiae* from Japan, Russia, and South Korea. The results of the analysis of both the mtDNA and microsatellite datasets indicated that the seven collection locations of *N*. *douglasiae* in the middle and lower Yangtze River drainage showed high genetic diversity, significant genetic differentiation and genetic structure, and stable population dynamics over time. Moreover, we found that the connections among tributaries rivers and lakes in the Yangtze River drainage were important in maintaining gene flow among locations that *N*. *douglasiae* inhabits. An understanding of the genetic structure and diversity of a widespread species like *N*. *douglasiae* could be used as a surrogate to better understand the populations of other freshwater mussel species that are more rare in the Yangtze River drainage. At the same time, these results could provide a basis for the protection of genetic diversity and management of unionid mussels diversity and other aquatic organisms in the system.

## Introduction

Freshwater mussels (Bivalvia: Unionida) are one of the most important faunas in freshwater ecosystems, for their potential to enhance biodiversity and ecosystem functioning (e.g., nutrient cycling and creating habitat for other benthic organisms)[1–4]. However, freshwater mussels are one of the most critically endangered faunal groups and are globally in decline[1,5]. Freshwater mussels also have an unusual life cycle, requiring a host fish species for larval (glochidia) development and dispersal[6]. This complex life cycle has the potential to drive genetic isolation between populations[7]. The obligate parasitic life stage may lead to phylogeographic patterns of conservation importance.

The middle and lower reaches of the Yangtze River are a center for freshwater mussel diversity in East Asia with 68 described species[8–11]. However, in these regions, more than 80% of freshwater mussels are considered to be threatened or near threatened, and the dominant taxa of bivalves have shifted from large-sized unionids to the small-sized corbuculid clams (Cyrenidae)[12–13]. The decline of freshwater mussels diversity and abundance in the middle and lower reaches of the Yangtze River may be attributed to a variety of threats[8–10,12–14]: 1) fluctuations in water levels in river and lake levels coinciding with wet and dry seasons have become more extreme as a result of dam construction in the watershed and possibly due to human-induced climate change–these water level fluctutations may be very important in structuring habitat, and thus population and assemblage structure of unionid mussels; 2) the construction of large-scale impoundments and sand mining from the river benthos has resulted in destruction of habitats and hindered the abilities of host fish (and glochidia from mussels) to disperse[15]; 3) overharvesting of fish (potentially) acting as hosts for mussels may be affecting the negatively affecting survivorship of unionids; and 4) commercial mussel harvest operations for the production of pearls and buttons is affecting many unionid species. These threats are causing delines in the abundance and distribution of unioinid species in the Yangtze drainage and may be fragmenting the historically more continuous habitats and populations. Due to these ongoing threats and documented declines in mussel populations, gaining an understanding of phylogeography, population structure, and genetic diversity is important for the conservation and management of unionid species in Yangtze River drainage[9–10,12]. Information on the genetic diversity and structure of natural populations is needed to inform resource managers on how to best mimic natural populations when restoration efforts are implemented. Protecting freshwater mussels in the Yangtze River has become especially important, and conservation and restoration projects should be prioritized to areas with higher genetic diversity when possible and the protection of unique (or endemic) diversity and lineages should they be discovered using genetic analyses[4,16].

*Nodularia douglasiae* (Griffith & Pidgeon, 1833; Bivalvia: Unionidae) is a relatively common and widespread freshwater mussel in the middle and lower reaches of the Yangtze River drainage, China; the Korean Peninsula; eastern Russia; and in Japan and the Sakhalin islands[1,3,17–18]. The large distribution and relatively high abundance of *N*. *douglasiae* make it an ideal species for understanding general patterns of freshwater mussel genetic structure in east Asia. Studies on common and widespread unionid species have previously been used as surrogates for better understanding genetic patterns in rare species[19–21]. Conservation programs are an integral part of freshwater mussel recovery plans, highlighting the need for large-scale assessment of freshwater mussel genetic structure and diversity[22]. Using this mussel species, the genetic structure of a common and widespread species will assist in the understanding of how drainage patterns have changed over time, and help in making inferences regarding the genetic structure among populations of rarer and/or narrowly distributed species[23–24]. In recent years, habitat destruction and human interference caused by large-scale construction projects [25], have threatened aquatic biodiversity, including freshwater mussels, throughout the distribution of *N*. *douglasiae*, making it a potentially important sentinel and surrogate species for rare and imperiled unionids in the region. Much of the distribution of *N*. *douglasiae* (e.g., the Yangtze River drainage) has numerous natural and anthropogenic features that may have shaped the genetic structure and diversity of this species[25].

Analyses of mitochondrial DNA sequence data provide estimates of the phylogenetic relationships and population evolution in unionid mussels[26–28]. Microsatellites or simple sequence repeats (SSR) are useful markers for the study of deeper genetic diversity patterns in freshwater mussels because of their co-dominance, high mutation rate, and ease of scoring[24, 29–31]. Combining analyses of mtDNA sequence data and microsatellite genotypes can help to reveal both the course-scale and fine-scale evolutionary history and genetic structure of a species. This study is the first to study the genetic structure and diversity of *N*. *douglasiae* in the middle and lower reaches of the Yangtze River, and among the first broad-scale studies for any freshwater mussel in the region. Given the historically interconnected nature of the Yangtze River and its tributary rivers and lakes, we hypothesize that the analyses will reveal high levels of genetic diversity and relatively little genetic structure with high levels of gene flow across sampled locations. This study will provide a basis for the protection and management of diversity in unionid mussels in this large river watershed.

## Materials and methods

### Ethics statement

All necessary permits were obtained for the described field studies from the Yangtze River Fishery Administration of China. The handling of mussels was conducted in accordance with the guidelines on the care and use of animals for scientific purposes set by the Institutional Animal Care and Use Committee (IACUC) of Nanchang University, Jiangxi, China.

### Sample collection and DNA extraction

**Fig 1:**
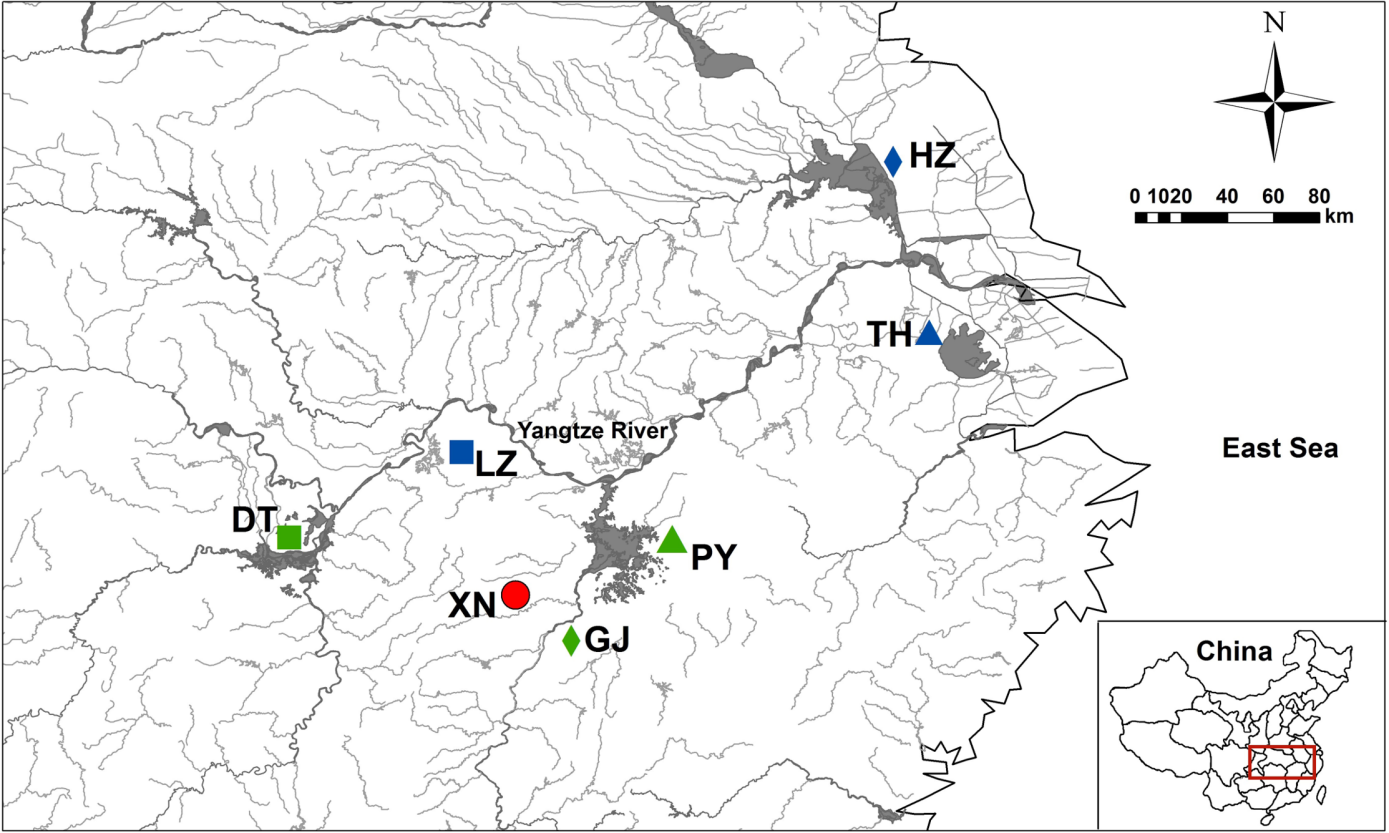
Collection locations for *N*. *douglasiae* in the middle and lower Yangtze River drainage. LZ: Liangzi Lake; DT: Dongting Lake; PY: Poyang Lake; GJ: Gan River; XN: Xiannv Lake; HZ: Hongze Lake; TH: Taihu Lake. Collection location symbol colours correspond to Fig 6 (K = 3).

Specimens of *N*. *douglasiae* (n = 197) were collected in 2014 and 2016 from Poyang Lake (PY), Donting Lake (DT), Xiannv Lake (XN), Gan River (GJ), Liangzi Lake (LZ), Hongze Lake (HZ), Taihu Lake (TH) in the middle and lower reaches of Yangtze River, China (Table 1 and Fig 1). Tissues of individual specimens were preserved in 95% ethanol and stored at -20°C until DNA extraction. Specimens were deposited in the Nanchang University Museum and assigned accession numbers (NCUXN1-Song-2014~NCUXN8-Song-2014, NCULZ1-Liu-2015~NCULZ8-Liu-2015, NCUPY21-Liu-2015~NCUPY28-Liu-2015, NCUDT1-Liu-2015~NCUDT8-Liu-2015, NCUHZ1-Liu-2016~NCUHZ8-Liu-2016, NCUTH1-Liu-2016~NCUTH8-Liu-2016, NCUGJ1-Liu-2016~NCUGJ16-Liu-2016). The genomic DNA was extracted from mantle tissue using the TINAamp Marine Animals DNA Kit. Concentration and quality of extracted DNA were estimated using a Nanodrop 2000 (Thermo Scientific) and agarose gel electrophoresis.

**Table 1:** Sample sizes and distribution information of *N*. *douglasiae* collected from the middle and lower Yangtze River drainage.

Collection location	Site code	Latitude	Longitude	Sample size Microsatellite genotypes	Sample size mtDNA COI sequences
Dongting Lake	DT	N29.126	E113.022	34	8
Poyang Lake	PY	N28.883	E116.292	32	8
Gan River	GJ	N28.663	E115.878	32	16
Xiannv Lake	XN	N27.733	E114.804	29	8
Hongze Lake	HZ	N33.298	E118.887	30	8
Taihu Lake	TH	N31.433	E120.331	30	8
Liangzi Lake	LZ	N30.256	E114.590	10	8

### PCR amplification

#### Mitochondrial DNA amplification

The cytochrome c oxidase subunit-I (COI) primer: the forward primer sequence was LCO22me2 (5′-GGTCAACAAAYCATAARGATATTGG-3′), the reverse primer sequence was HCO700DY2 (5′-TCAGGGTGACCAAAAAAYCA-3′). Each primer pair was screened for reliable amplification using 64 individuals of *N*. *douglasiae*. The PCR reaction was carried out in a 25 μL volume containing 12.5 μL 2x Taq PCR MasterMix (TianGen); 8.5 μL ddH_2_O; 1.0 μL of 10 μM forward primer; 1.0 μL of 10 μM reverse primer; and 2 μL genomic DNA (about 100 ng/μL). PCR amplifications were conducted with the following touchdown thermal cycling program: an initial denaturation at 94°C for 2 min, followed by 35 cycles of 94°C for 1 min, annealing temperature of 50°C for 1 min, 72°C for 1 min, and a final extension at 72°C for 7 min. The PCR products were electrophoresed on a 1% agarose gel to confirm successful amplification and then purified using an EZ-10 Spin Column PCR Product Purification Kit (Promega, Madison, WI). The purified DNA was then sequenced on an ABI 3730XL DNA Analyser (Applied Biosystems, Carlsbad, CA).

#### Microsatellite locus amplification

We used 13 primer sets for PCR amplification of microsatellite loci (S1 Table). A description of the development and initial screening of 14 new microsatellite loci (12 of which were used in this study) for *N*. *douglasiae* is included in Supporting Information (S1 File). One of the 13 loci (scasst17) used was developed for *Solenaia carinata* (Heude, 1877)[32]. Each primer pair was screened for reliable amplification using 197 individuals of *N*. *douglasiae*. The PCR reaction was carried out in a 20 μL volume containing 10 μL 2x Taq PCR MasterMix (TianGen); 6.4 μL water; 0.6 μL of 10 μM HEX-, TAMRA-, or 6-FAM-labeled M13 universal primer; 1.0 μL of 10 μM forward primer with an M13 tag on the 5′ end; 1.0 μL of 10 μM reverse primer; and 2 μL genomic DNA (about 50 ng/μL). PCR amplifications were conducted with the following touchdown thermal cycling program: an initial denaturation at 94°C for 5 min, followed by 10–15 cycles of 94°C for 30s, locus-specific annealing temperature ranging between 52°C and 65°C for 45 s, 72°C for 45 s, at the same time, 20 cycles of 94°C for 30 s, locus-specific annealing temperature 53°C for 45 s, 72°C for 45 s, and a final extension at 72°C for 10 min. All loci were run separately on an ABI3730 automated sequencer and alleles were scored using a TAMRA-labeled size standard[33] using GENEMAPPER v. 3.7 (Applied Biosystems).

### Data analyses

#### Mitochondrial DNA

The sequences of the mtDNA COI fragment in 64 samples were aligned using Clustal X1.81[34]. DNASP 5.0 [35] was used to analyse nucleotide composition, number of polymorphic sites (S), average numbers of pairwise nucleotide differences, haplotype diversity (Hd) and nucleotide diversity (π) for each collection location.

To test the monophyly of the *N*. *douglasiae* COI haplotypes from the middle and lower Yangtze drainage, a phylogenetic analysis using Bayesian inference was performed using MRBAYES v.3.2.2 [36]. The initial model of evolution (HKY+G) was determined by comparing 24 models of evolution in MRMODELTEST v.2.2[37]. MRBAYES was run using 3,000,000 generations and six concurrent Markov Chains and 2 hot chains sampled at intervals of every 100 generations for a total of 30,000 trees. A 25% burn-in (7500 trees) was used to ensure stationarity of the log likelihood values. In addition to the 37 COI haplotypes from the middle and lower Yangtze, COI sequences from other putative *N*. *douglasiae* from Russia, Japan, and South Korea [18] were included (S4 Table). Also included in the phylogenetic analyses were COI sequences for *N*. *nuxpersicae* [11], *N*. *nipponensis* [18], and *N*. *sinuolata* [18] (S4 Table). As outgroups for the *Nodularia* dataset, COI sequences available in GenBank from 10 species were used (S4 Table).

To visualize the relationships among the *N*. *douglasiae* COI haplotypes from the middle and lower Yangtze, a haplotype network was constructed using a TCS algorithm[38] in POPART[39] with a 95% connection limit and gaps defined as missing data.

Patterns of genetic structure in the COI dataset were evaluated using a hierarchical analysis of molecular variance (AMOVA). The AMOVA was used to partition variance components to populations and to individuals within each collection location, where 1000 permutations were performed to test the significance of each pairwise population comparison. A test for isolation-by-distance was conducted by testing the significance of a correlation between pairwise Nei’s D (calculated using ARLEQUIN[40]) and geographic distance among sampling locations. The correlation computations between pairwise genetic and geographic distances between populations were analysed using a Mantel test[41]. Geographic distance was meansured among collection sites by measuring distances following waterways in ArcMap GIS (ESRI).

Tajima’s D and Fu’s Fs tests were conducted through Arlequin 3.5[40], to examine deviations from neutrality. DNASP 5.0[35] was used to analyse mismatch distribution analysis (MDA). A Bayesian Skyline Plot (BSP)[42] analysis was computed in BEAST 1.4.7[43]. The BSP was used to reconstruct the effective population size fluctuations since the time of the most recent common ancestor (TMRCA). MCMC was run for 500 million steps, with sampling every 1000 generations and following a ‘burn-in’ of the initial 10% cycles. Inspections of the results and construction of the BSP were conducted using TRACER 1.5[44]. The fit of the constant size population model and Bayesian Skyline coalescent models to dataset was assessed using the Model Comparison function in TRACER 1.5.

#### Microsatellite DNA

The number of alleles (*N*_*A*_), the effective number of alleles (*N*_*E*_), observed heterozygosity (*H*_*O*_) and expected heterozygosity (*H*_*E*_), and tests for deviation from Hardy–Weinberg Equilibrium (HWE) were calculated using POPGENE, v1.32[45]. CERVUS 3.03[46] was used to calculated polymorphism information content (PIC). Using MICRO-CHECKER v. 2.2.3[47] to detect possible null alleles from each collection location.

In order to detect any recent genetic bottlenecks (within 2*N*_e_− 4*N*_e_ generations), four tests with varying degrees of sensitivity were conducted using BOTTLENECK v. 1.2.02[48]. Wilcoxon sign rank tests were carried out using three models of evolution: the infinite alleles model (IAM), two-phase model (TPM), and stepwise mutation model (SMM). A mode-shift test was conducted to identify significant changes in allelic frequency caused by a genetic bottleneck.

Using STRUCTURE v. 2.3.3[49] population structure was assessed in the study area. Ten iterations, allowing for admixture among genetic groups (*K*) and assuming correlated allele frequencies, were run for each value of *K* (number of clusters) which was defined by the number of collection locations for each species: the maximum value of K was calculated by adding 3 to the number of collection locations (i.e., K = 1–10) to allow detection of substructure within sampling locations. Each trial used an initial burn-in period of 200,000 replicates, followed by an additional 200,000 replicates after burn-in to ensure stationarity. To determine optimal solutions for potential numbers of genetic groups (K) within each species[50], calculating ΔK from STRUCTURE output in combination with the log likelihood of the solution for each value of K using STRUCTURE HARVESTER v. 0.6.8[51]. To further evaluate and visualize the geographic genetic structure among collection locations, a principal coordinates analysis (PCoA) was conducted using GenAlEx 6.5[52] to ordinate genetic distance estimates[53] calculated for the genotypic data of individuals used.

An analysis of molecular variance (AMOVA)[54] was run using GenAlEx to test the statistical significance of genetic divergences within and among collection locations in each population. Pairwise analyses of genetic divergence (*F*_ST_ and Jost’s *D*)[55] among sampling locations were calculated using GenAlEx. Geneflow was estimated by calculating number of migrants per generation (*N*_m_) using GenAlEx. A test for isolation-by-distance was conducted by testing the significance of a correlation between pairwise Nei’s D (calculated using GenAlEx) and geographic distance among sampling locations. The correlation computations between pairwise genetic and geographic distances between populations were analysed using a Mantel test [41]. Geographic distance was meansured among collection sites by measuring distances following waterways in ArcMap GIS (ESRI).

## Results

### Mitochondrial DNA

From the 64 sequenced individuals collected from 7 locations in the middle and lower Yangtze River drainage, 37 unique COI haplotypes were identified (GenBank Accession Nos. MG210495-MG210558). The Gan River had the greatest variation with 13 haplotypes, and the lowest was Poyang Lake with 4 haplotypes (Table 2). Haplotypes diversity values at each population varied between 0.857 and 0.975 (Table 2). The Gan River had the greatest haplotype diversity (0.975), and the lowest was Poyang Lake (0.857). Nucleotide diversity values ranged from 0.00726 to 0.04592 (Table 2). Poyang Lake had the greatest nucleotide diversity (0.04592), and the lowest was Liangzi Lake (0.00926).

**Table 2:** Descriptive statistics of COI for each collection site of *N*. *douglasiae* collection locations in the middle and lower reaches of Yangtze River.

Sample site	N	H	Hd	π
DT	8	7	0.964	0.01726
LZ	8	7	0.964	0.00926
PY	8	4	0.857	0.04592
GJ	16	13	0.975	0.02728
XN	8	6	0.929	0.03448
HZ	8	6	0.929	0.03777
TH	8	7	0.964	0.01392
Total	64	37	0.976	0.03192

Site codes as in Table 1. N = number sequenced, H = number of haplotypes, Hd = haplotype diversity, π = mean nucleotide diversity.

**Fig 2:**
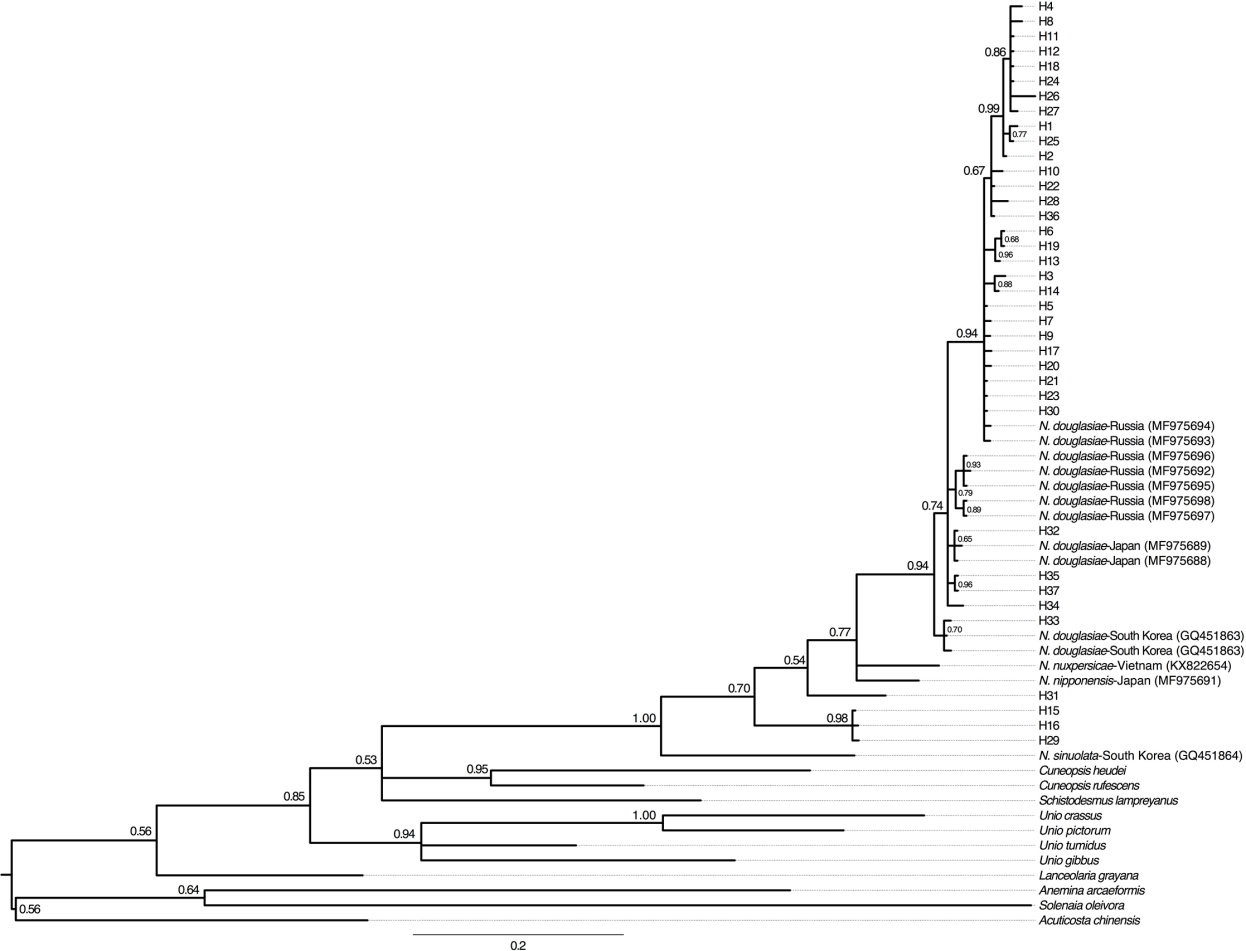
Phylogenetic tree of the COI fragment for *Nodularia* obtained using Bayesian Inference (BI). Support values represent BI posterior probabilities. Only support values above 0.50 are shown.

Phylogenetic analyses (Fig 2) showed strong support for the monophyly of *Nodularia* (posterior probability = 1.00). *Nodularia nuxpersicae* and *N*. *nipponensis* were found to nest within the 37 *N*. *douglasiae* haplotypes sequenced from the Yangtze and additional *N*. *douglasiae* COI sequences from Japan, Russia, and South Korea. The 37 COI haplotype sequences from the Yangtze drainage in combination with *Nodularia* COI sequences from GenBank formed three clades: three haplotypes restricted to Xiannv Lake, Poyang Lake, and the Gan River (H15, H16, and H29); a single haplotype found in two individuals from Hongze Lake (H31); *N*. *nuxpersicae*, *N*. *nipponensis* and the remaining 33 haplotypes formed the largest clade. *Nodularia sinuolata* from South Korea was sister to all of the remaining *Nodularia* COI seqeuences.

**Fig 3:**
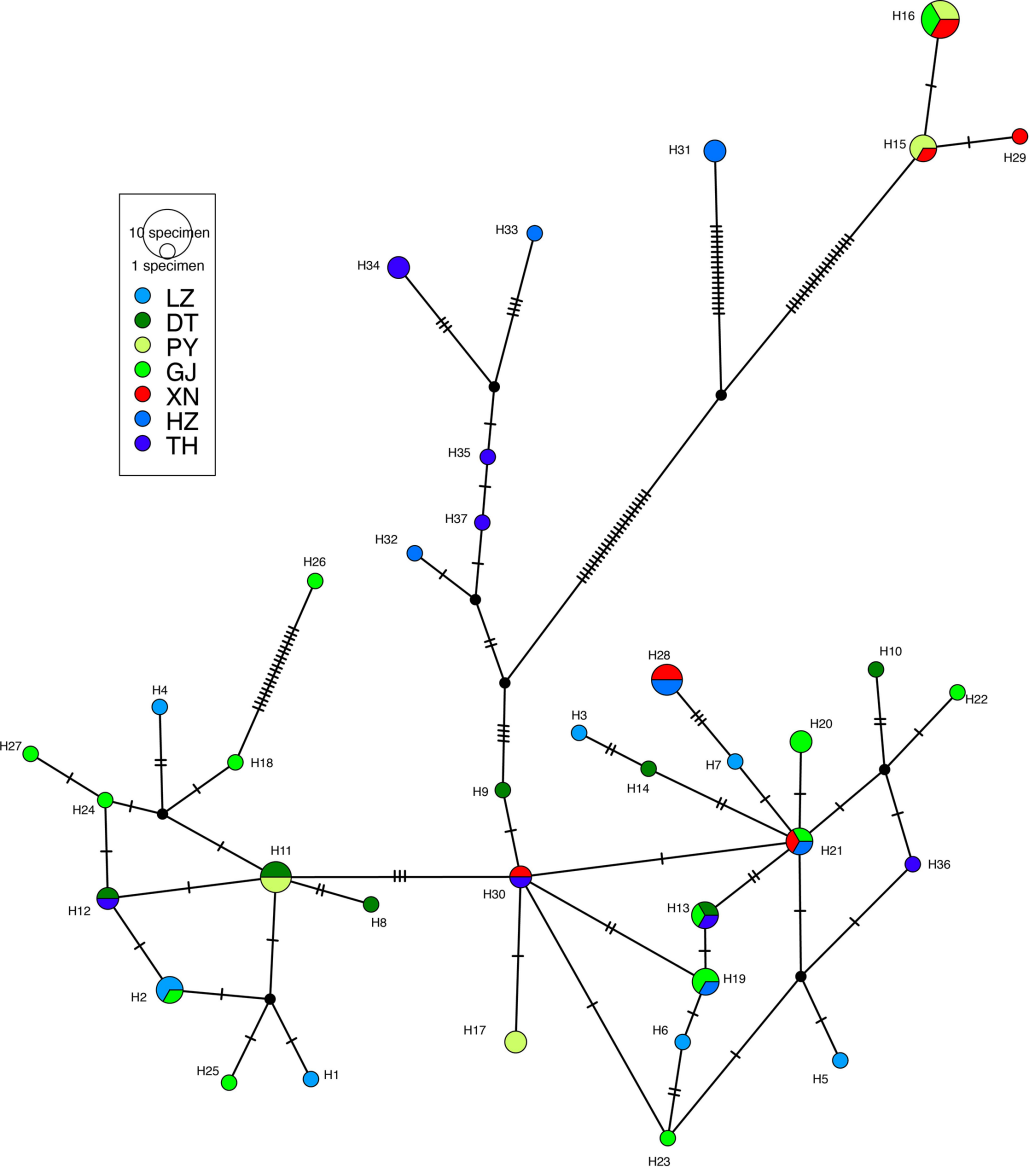
Haplotype network for *N*. *douglasiae* populations in the middle and lower reaches of Yangtze River. Each cross-hatched line represents one base-pair difference between haplotypes, black dots are inferred missing haplotypes, and haplotype frequency is relative to the size of the circle. Collection location codes as in Table 1. Colours used are in similar hues (blue, green, red) corresponding to the results of the STRUCTURE analysis (K = 3, Fig 6).

Using the TCS algorithm, POPART software produced a single haplotype network (Fig 3). The most frequent haplotype (H16) occurred in 6 individuals and was shared by individuals in the Gan River, Poyang Lake, and Xiannv Lake. Twenty-three haplotypes were rare and occurred in just a single individual. As seen in the phylogeny (Fig 2), for the most part, the haplotypes show little geographic structure. However, a unique group of haplotypes (H32, H33, H34, H35, and H37) were found in Hongze Lake and Taihu Lake, the easternmost collections.

The AMOVA results showed that 11.99% of the total genetic variance was among the seven collection locations, and among sampling location differentiation was significant (overall *F*_ST_ = 0.1874, *p*<0.001, Table 3). Pairwise *F*_ST_ ranged from -0.025 to 0.393 among the collection locations (Table 4).

**Table 3:** Analysis of molecular variation (AMOVA) calculated from COI mtDNA sequences for *N*. *douglasiae* from seven collection locations in the middle and lower reaches of Yangtze River.

Source of variation	df	Sum of squares	Variance components	Percentage of variation
Among populations	6	158.375	1.979	11.99
Within populations	57	489.250	8.583	88.01
Total	63	647.625	10.562	

All F-statistics were statistically significant (*p*<0.001).

**Table 4:** Analysis of genetic differentiation coefficient (*F*_st_) calculated using COI mtDNA sequence data among seven collection locations of *Nodularia douglasiae* from the middle and lower Yangtze River drainage.

	LZ	DT	PY	GJ	XN	HZ	TH
LZ							
DT	-0.026						
PY	**0.357**	0.360					
GJ	-0.022	-0.014	0.186				
XN	**0.383**	**0.393**	**0.120**	**0.212**			
HZ	**0.172**	**0.182**	**0.150**	**0.076**	0.152		
TH	**0.197**	**0.243**	**0.348**	**0.096**	**0.365**	0.087	

Bold type indicates statistical significance after Bonferonni correction (α = 0.002381).

Genetic differentiation as represented by pairwise genetic distance values among the seven collection locations was not correlated with geographic water distance indicating that more geographically distant site combinations did not produce higher levels of genetic differentiation (*p* = 0.5020).

**Fig 4:**
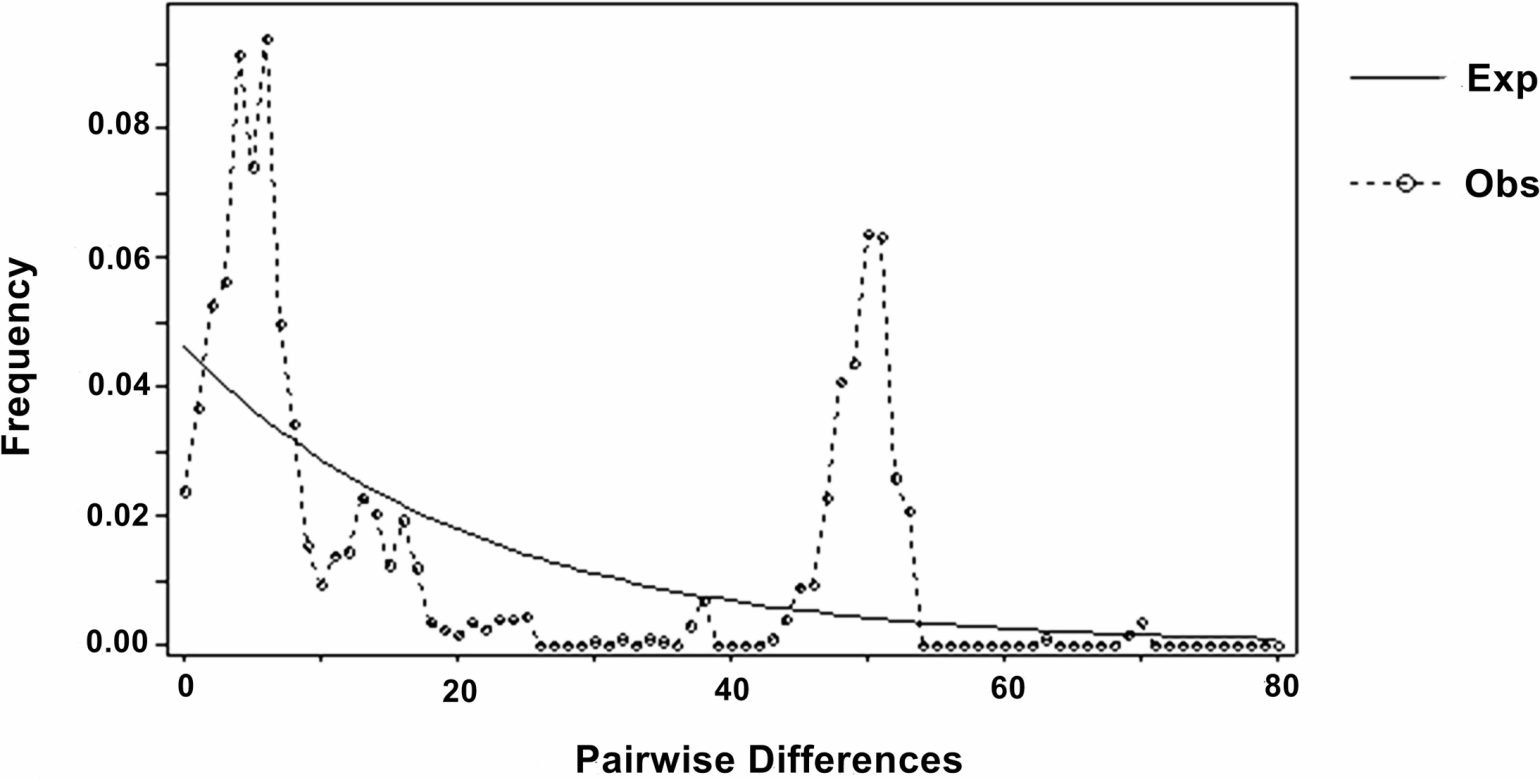
Mismatch distribution analysis (MDA) for *N*. *douglasiae* collection locations in the middle and lower reaches of Yangtze River.

**Fig 5:**
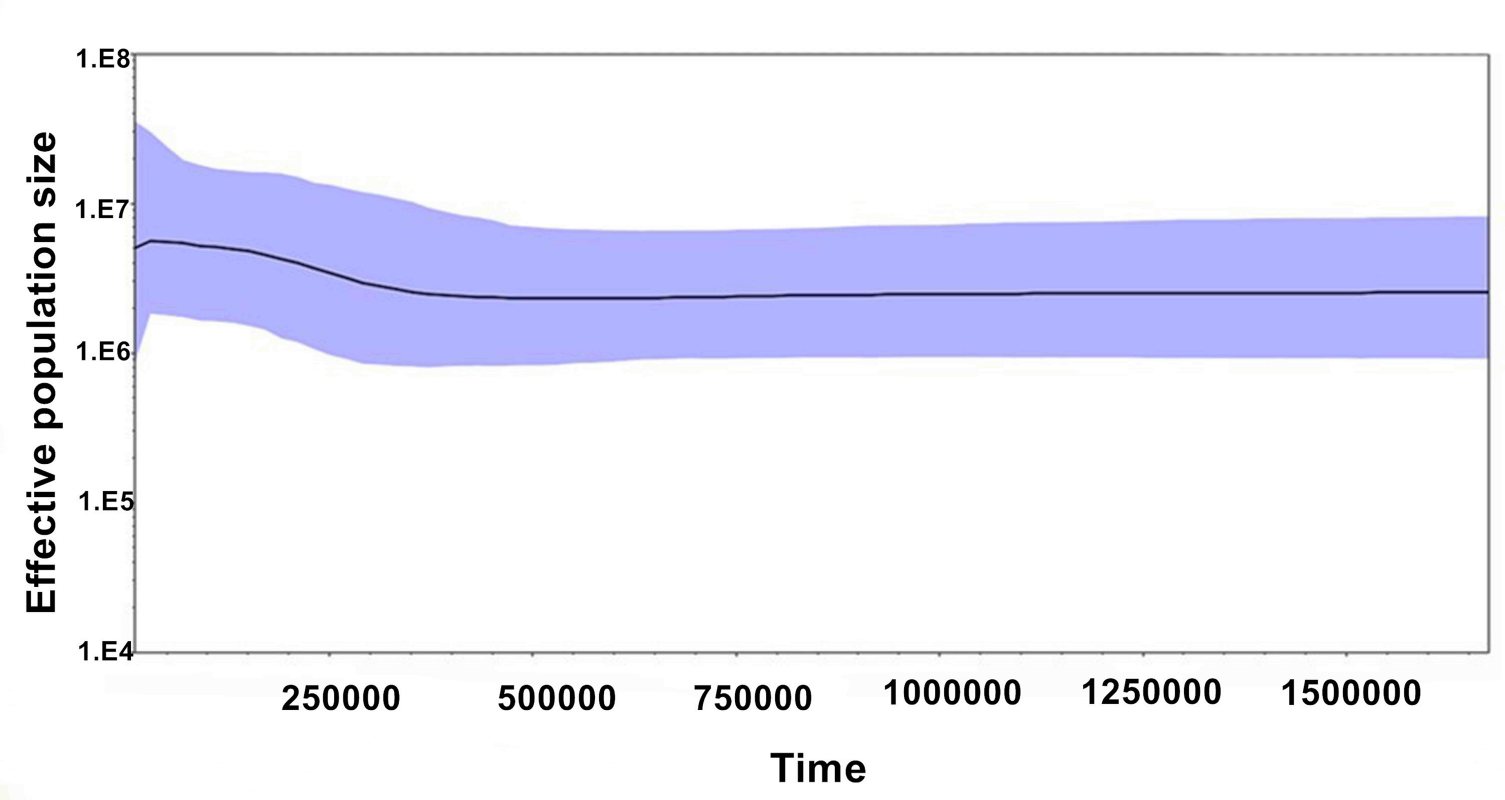
Bayesian skyline plot for *N*. *douglasiae* from the middle and lower Yangtze River drainage reconstructing the population size history using an evolutionary rate of 2.0 × 10^−8^ substitutions/site/year.

The mismatch distribution of analysis pairwise differences was significantly different from the expected distribution of the expanding population model (Fig 4). Similarly there was a lack of statistical significance of Tajima’s D test (*p*<0.01), and non-significant Fu's FS (*p*<0.01). Moreover, when all samples were pooled together, Tajima’s D and Fu's FS test were not significant (*p*<0.01, Table 5). Additionally, the BSPs showed that *N*. *douglasiae* has had a stable historical population size with a small recent expansion event occurring between 250,000 and 300,000 years (Fig 5). However, the model comparison analysis showed that constant population size was the best fit for the model to the data set, suggesting that there was not much support for the recent expansion trend.

**Table 5:** Neutrality tests for *N*. *douglasiae* collection locations in the middle and lower reaches of Yangtze River (P<0.01). Site codes as in Table 1.

Collection locations	Sample size	Pi(%)	Tajima's D	Fu's FS	Mismatch distribution	
					P_SSD_	P_RAG_
DT	8	4.679	-0.339	-2.067	0.800	0.950
LZ	8	5.964	-0.171	-1.507	0.350	0.280
PY	8	29.571	2.406	8.293	0.600	0.440
GJ	16	17.567	-0.955	-0.797	0.580	0.890
XN	8	28.642	2.305	3.225	0.120	0.920
HZ	8	24.321	0.790	2.842	0.230	0.930
TH	8	8.964	0.844	-0.666	0.400	0.790
All collection locations	64	20.559	-0.194	-3.556	0.360	0.710

### Microsatellite DNA

A total of 197 individuals of *N*. *douglasiae* were successfully genotyped for all 13 microsatellite loci. The results showed that 434 alleles were detected among the seven collection locations. The number of alleles (*N*_A_) at each sampling location ranged from 3.231 to 5.692, the effective number of alleles (*N*_E_) ranged from 2.223 to 2.538, observed heterozygocity (*H*o) ranged from 0.454 to 0.522, expected heterozygosity (*H*_E_) ranged from 0.442 to 0.531, and polymorphic information criterion (PIC) ranged from 0.435 to 0.477 (Table 6). Deviations from HWE were found at only 8 of 91 locus-collection site combinations loci after a Bonferonni correction and were not consistently found at any site or locus (S2 Table). Significant tests for null alleles occurred in 8 of the loci used, however the estimated null allele frequencies were generally low ranging from 0.000 to 0.2792 at any given collection location-locus combination (S3 Table). The only locus where null alleles were consistently detected at levels that could potentially affect the outcomes of population-level results[56–57] was locus Udo14. Analyses (e.g., STRUCTURE) were rerun without locus Udo14 and were found to give similar patterns to the results run with this locus, thus locus Udo14 was kept for all analyses.

**Table 6:** Population genetic parameters in seven populations of *N*. *douglasiae* in the middle and lower Yangtze River drainage calculated using 13 microsatellites.

Genetic parameters	LZ	DT	PY	GJ	XN	HZ	TH
N	10	34	32	32	29	30	30
N_A_	3.231	5.538	5.077	5.692	4.231	4.769	4.846
N_E_	2.223	2.538	2.377	2.446	2.231	2.408	2.246
H_E_	0.481	0.531	0.490	0.501	0.442	0.502	0.478
H_O_	0.454	0.481	0.460	0.475	0.483	0.522	0.503
F_is_	0.046	0.194	0.147	0.168	-0.078	-0.008	-0.081
PIC	0.435	0.477	0.440	0.476	0.436	0.473	0.460

N: number genotyped; N_A_: the number of alleles; N_E_: the effective number of alleles; H_E_: expected heterozygosity; H_O_: observed heterozygosity; F_is_:fixation index; PIC: polymorphic information content. Collection location codes as in Table 1.

Wilcoxon tests showed evidence for a recent genetic bottleneck at all of the locations except for Liangzi Lake using the SMM model (Table 7). The Gan River also showed a significant bottleneck using the TPM model (p<0.05).

**Table 7:** Results of tests for genetic bottlenecks in *N*. *douglasiae* from seven populations in the middle and lower reaches of Yangtze River using Wilcoxon tests with three different models of evolution and a mode-shift test. Collection location codes as in Table 1.

Population	IAM	TPM	SMM	Mode-shift
LZ	0.8926	0.6848	0.2439	normal L-shaped
DT	0.8394	0.4973	0.0085*	normal L-shaped
PY	0.5418	0.1909	0.0040*	normal L-shaped
GJ	0.3757	0.0479*	0.0012*	normal L-shaped
XN	1.0000	0.2163	0.0215*	normal L-shaped
HZ	0.7354	0.3054	0.0085*	normal L-shaped
TH	0.4548	0.0803	0.0067*	normal L-shaped

*Significant evidence of a recent genetic bottleneck (*p*<0.05).

**Fig 6:**
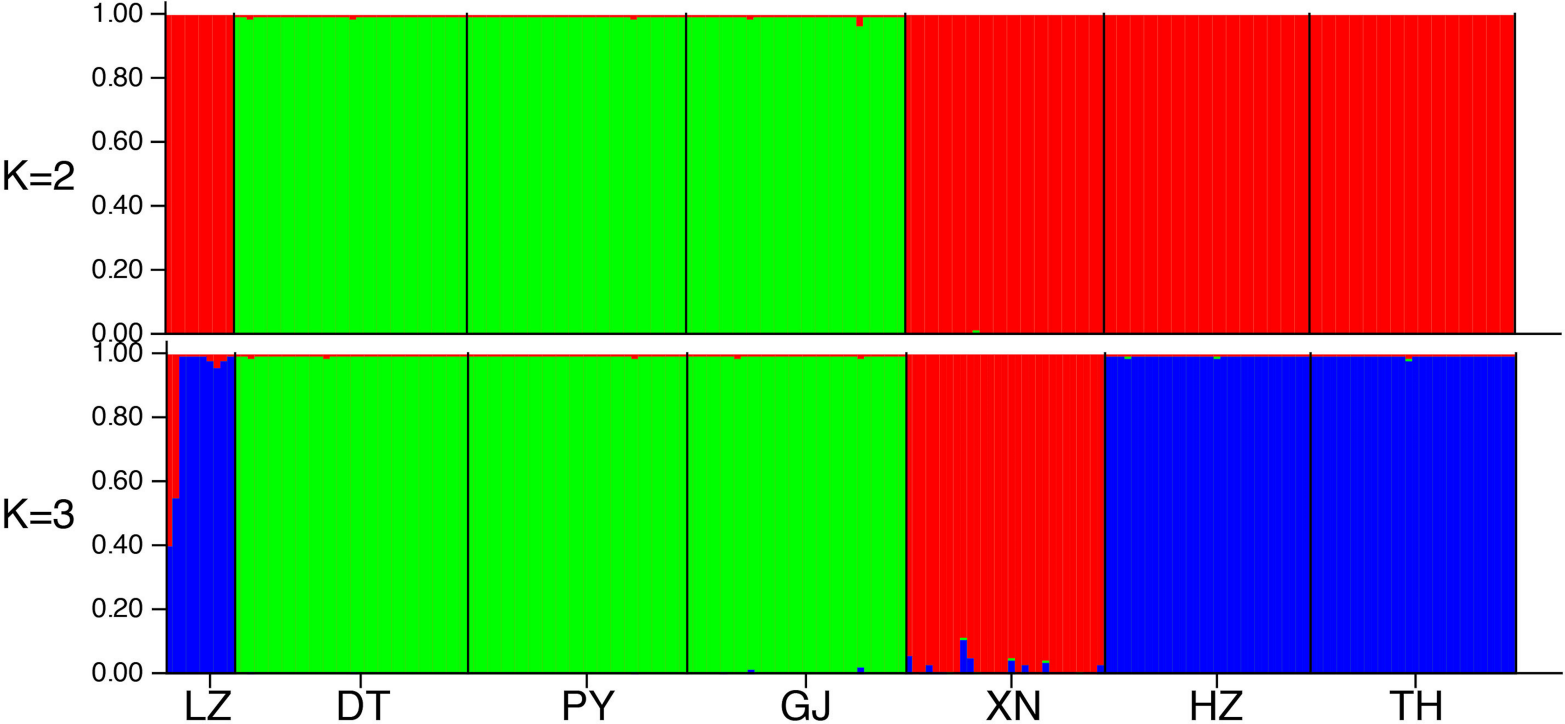
STRUCTURE bar plots for seven *N*. *douglasiae* collection sites in the middle and lower reaches of Yangtze River. STRUCTURE runs were completed without *a priori* populations assigned, admixture and correlated alleles were assumed, The most probable number of populations was K = 2 using the Evanno et al.^[^^33^^]^ method, but K = 3 had slightly higher ln likelihood scores. Collection location codes are as in Fig 1.

**Fig 7:**
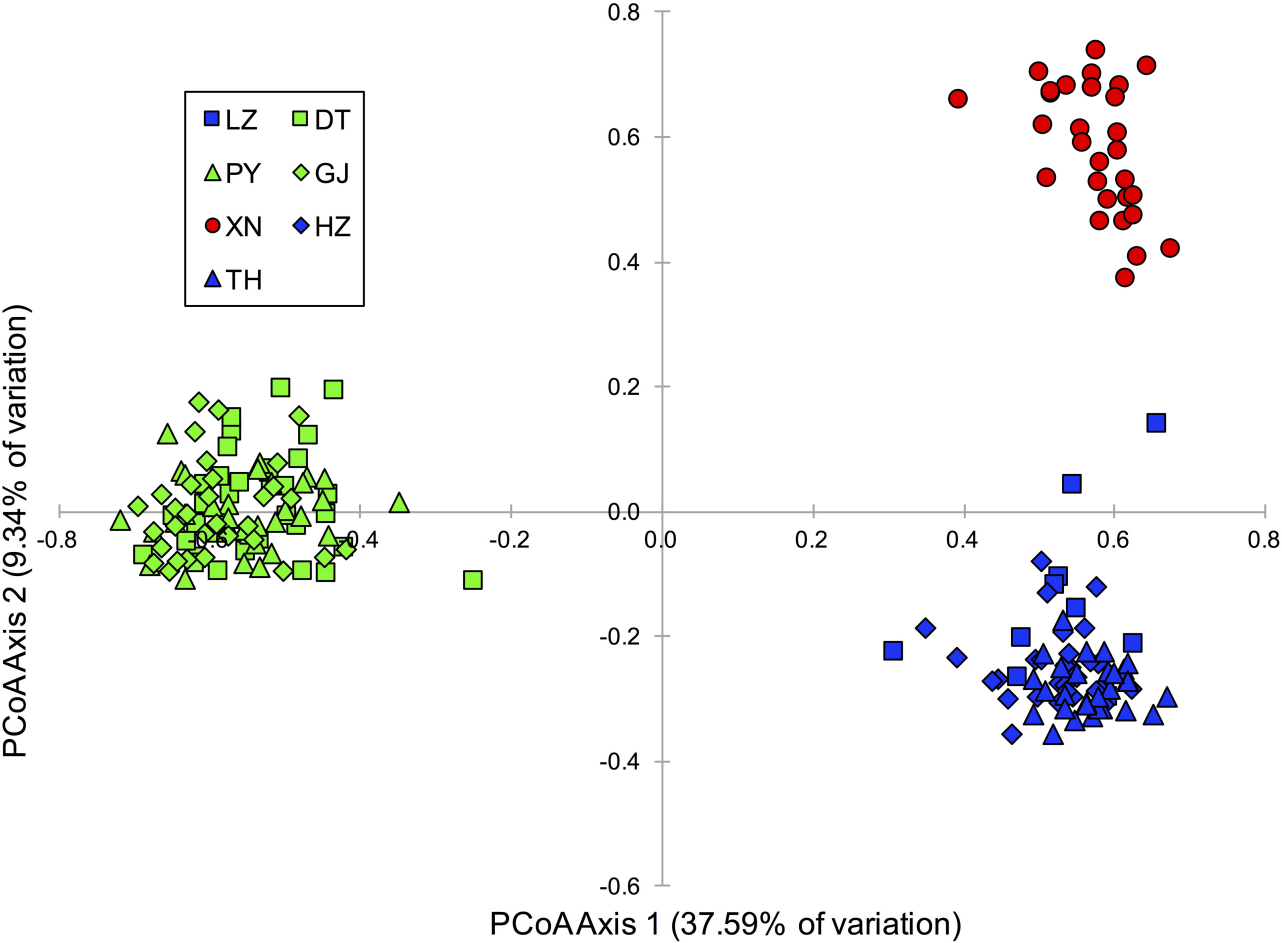
Principal Coordinates Analysis (PCoA) based on multilocus genotypes of individuals of *N*. *douglasiae* from seven collections sites in the middle and lower reaches of Yangtze River. Collection location codes and colours are as in Fig 1.

Using the data generated from the STRUCTURE analysis, the Evanno et al.[50] ΔK method indicated that K = 2 was the most likely (S1 Fig). Under K = 2, Dongting Lake, Poyang Lake, and the Gan River formed one genetic population and Hongze Lake, Taihu Lake, Liangzi Lake, and Xiannv Lake formed a second genetic population. While, K = 2 was most probable using the Evanno et al.[50] method, K = 3 had a slightly higher log-likelihood score (S1 Fig). With K = 3, Xiannv Lake became a distinct group (Fig 6). The PCoA showed a similar pattern of genetic structure (Fig 7) to the STRUCTURE analysis, with two clusters appearing along axis 1 and Xiannv Lake showing differentiation along axis 2. The PCoA explained 46.9% of the genetic variation across the 13 microsatellite loci in the first two axes.

The AMOVA results showed that 36.0% of the total genetic variance was among the seven collection locations, and among sampling location differentiation was significant (*p*<0.0001, Table 8). Pairwise *F*_ST_ and Jost’s *D* was congruent with the pattern of genetic structure revealved by analyses done in STRUCTURE and the PCoA showed that genetic differentiation was significant was moderate to high among the seven collection locations with the exception of the comparisons among Dongting Lake, Poyang Lake, and the Gan River and between the Hongze Lake and Taihu Lake (Table 9). Using a Mantel test, genetic differentiation (*F*_st_ and Jost’s *D*) among the collection locations using microsatellites was not significantly correlated with pairwise *F*_st_ values calculated for the mtDNA (p>0.05). Estimates of gene flow (*N*_m_) were generally low (i.e., *N*_m_ <1) except for among Dongting Lake, Poyang Lake, and the Gan River and between the Hongze Lake and Taihu Lake (Table 10).

**Table 8:** Analysis of molecular variation (AMOVA) calculated using 13 microsatellite loci for *N*. *douglasiae* from seven populations in the middle and lower reaches of Yangtze River.

Source of variation	df	SS	Variance components	Percent variation (%)	F-Statistics
Among populations	6	562.2	1.63	36	*F*_st =_ 0.359
Among individuals within populations	190	588.7	0.18	4	*F*_is_ = 0.063
Within individuals	197	538.0	2.73	60	*F*_it_ = 0.399

All F-statistics were statistically significant (p<0.0001).

**Table 9:** Analysis of genetic differentiation coefficient (*F*st) (below diagonal) and Jost *D*_est_ (above diagonal) calculated using genotypes from 13 microsatellite loci among seven collection locations of *Nodularia douglasiae* from the middle and lower Yangtze River drainage. Bold type indicates statistical significance after Bonferonni correction (α = 0.002381).

	LZ	DT	PY	GJ	XN	HZ	TH
LZ		**0.652**	**0.662**	**0.691**	**0.317**	**0.094**	**0.088**
DT	**0.416**		0.007	**0.030**	**0.746**	**0.613**	**0.646**
PY	**0.438**	0.008		**0.034**	**0.760**	**0.616**	**0.653**
GJ	**0.461**	**0.036**	**0.044**		**0.787**	**0.653**	**0.687**
XN	**0.291**	**0.484**	**0.523**	**0.523**		**0.331**	**0.348**
HZ	**0.091**	**0.401**	**0.440**	**0.440**	**0.290**		0.016
TH	**0.096**	**0.435**	**0.474**	**0.474**	**0.320**	0.018	

**Table 10:** Estimated gene flow (*N*m) (below diagonal) calculated using genotypes form 13 microsatellite loci and among seven collection locations of *N*. *douglasiae* from the middle and lower Yangtze River drainage.

	LZ	DT	PY	GJ	XN	HZ	TH
LZ							
DT	0.351						
PY	0.320	30.465					
GJ	0.292	6.790	5.713				
XN	0.610	0.267	0.246	0.228			
HZ	2.508	0.373	0.350	0.318	0.613		
TH	2.349	0.325	0.303	0.277	0.532	13.402	

Genetic differentiation as represented by pairwise genetic distance values between all seven sample sites were shown to be not correlated with geographic water distance indicating that more geographically distant site combinations did not produce higher levels of genetic differentiation (p = 0.485).

## Discussion

The results of this study show clear genetic structure in *N*. *douglasiae* across the middle and lower Yangtze River drainage. Two clear genetic groups are revealed using analyses of microsatellite genotypes consisting of 1) Dongting Lake, Poyang Lake, and the Gan River and 2) Liangzi Lake, Xiannv Lake, Hongze Lake, and Taihu Lake. The pattern of genetic structure found using the mtDNA dataset is only partially congruent with the pattern revealed by the microsatellites. The main difference between patterns reveled by the microsatellite and mtDNA datasets was that specimens from Liangzi Lake grouped with specimens from Hongze Lake and Taihu Lake with the microsatellite data, but grouped more closely with Dongting Lake and the Gan River with the mtDNA dataset. The overall resulotion of the pattern of geographic structure among the collection locations was fairly poor and inconsistent using the mtDNA dataset. This poor resolution and inconsistency may be a result of the very high haplotypic diversity among the specimens used (37 haplotypes from 64 individuals) and relatively small sample size of the mtDNA dataset.

The microsatellite dataset is robust and of high quality with few loci out of Hardy-Weinberg equilibrium and relatively low numbers of null alleles predicted to be present in the dataset. The estimated null allele frequencies were generally below thresholds that would impact the results or interpretations of population-level analyses[56–57]. Null alleles in microsatellite datasets are frequently encountered and appear common in bivalves[58–59].

### Evolutionary history and genetic structure

The geographic structure among the sampling locations using the mtDNA dataset was somewhat ambiguous. This ambiguity was likely a result of the high haplotype diversity (37 haplotypes), but relatively small sample size (n = 64). The COI phylogeny did not resolve *N*. *douglasiae* to be monophyletic due to the inclusion of *N*. *nuxpersicae* from Vietnam and *N*. *nipponensis* from Japan. Klishko et al.[18] found that *N*. *nipponensis* was sister to the COI sequences of *N*. *douglasiae* that they used in their analysis and thus chose to maintain *N*. *nipponensis* as a valid taxon. The phylogeny resolved in this study found that *N*. *nipponensis* and *N*. *nuxpersicae* were nested within the *N*. *douglasiae* COI sequences from the Japan, Russia, South Korea[18], and the Yangtze drainage (from this study); there are two possible explanations for this: (1) that *N*. *douglasiae; N*. *nipponensis*; *N*. *nuxpersicae*; the clade of haplotypes H15, H16, and H29; and haplotype H31 are each distinct species or (2) that each of these are divergent lineages of *N*. *douglasiae* and that *N*. *nipponensis* and *N*. *nuxpersicae* can be synonymized into *N*. *douglasiae*. The latter explanation is also supported by the pattern of genetic structure revealed by the analyses of the microsatellite dataset (see below). As a result of the phylogenetic analysis of the mtDNA dataset and the microsatellite analyses, only *N*. *douglasiae* and *N*. *sinuolata* appear to be valid species. Additional studies across the distribution of *Nodularia* in east Asia appear warranted to determine if *Nodularia* is a single species or if multiple (possibly cryptic species) exist.

Given that the *N*. *douglasiae* COI lineages from the middle and lower Yangtze were distributed across several collection locations, it seems that *N*. *douglasiae* is a single species in the study area. One lineage was found in Poyang Lake, the Gan River, and Xiannv Lake, and Hongze Lake (H15, H16, H29, H31; Fig 3), while the second highly diverse lineage was broadly distributed across all collection locations. The haplotype network results (Fig 3) indicated that haplotype diversity was high, but that closely related haplotypes were broadly distributed across most of the sampling locations. An AMOVA of the COI sequence data showed that genetic differentiation was significant among many of the collection locations and that some sampling locations were significantly differentiated from one another. The general pattern of haplotype differentiation among sampling locations indicated the following groups: Hongze Lake and Taihu Lake; Liangzi Lake and Dongting Lake; and Ponyang Lake and Gan River.

Analyses of microsatellite dataset shows that high levels of genetic differentiation exist among the collection locations of *N*. *douglasiae*[60], but do not support the existence of more than one species of *Nodularia* in the study area. The analyses of the microsatellites were much better than the results of the mtDNA sequence data at resolving geographic structure among the collection locations. Despite this pattern, genetic differentiation was low and gene flow was high among Dongting Lake, Poyang Lake, and the Gan River and among the Hongze Lake, Taihu Lake, and Liangzi Lake. The Xiannv Lake collection location was most similar to the Hongze Lake, Taihu Lake, and Liangzi Lake, but was found to be genetically distinct. This pattern of overlap can also be seen in the STRUCTURE plots (Fig 6) and the PCoA (Fig 7). All of the analyses of genetic structure, genetic differentiation, and gene flow were congruent. The pattern of genetic structure revealed by the analyses of the microsatellites is geographic in nature and suggests that the divergent COI lineages freely interbreed as the lineages were found to occur across the groups revealed by the microsatellites (e.g., Xiannv Lake, Poyang Lake, and Gan River).

The results suggest that the connectivity of rivers and lakes in the Yangtze River drainage was very important for dispersal in *N*. *douglasiae*. Historically, all seven collection locations were hydrologically connected, however, habitat alterations as a result of dam construction, dredging for canals and sand/gravel mining, and a major increase in urbanization in the last 100 years have had considerable effects of the connectivity among the mainstem of the Yangtze River and its tributaries rivers and lakes[15]. Of the areas sampled in this study, only Poyang Lake (and the Gan River) and Dongting Lake continue to have direct and natural connections to the mainstem of the Yangtze River (i.e., no dams)[12, 61]. The Gan River is the largest river running from north to south in Jiangxi Province, China, flowing into Poyang Lake, and is the seventh largest tributary of the Yangtze River. In 1958, a dam was constructed at the outlet of Xiannv Lake that blocked upstream connectivity from the Gan River (and Poyang Lake)[62]. Between 1942 and 1963, hydrologic connectivity between Liangzi Lake and the Yangtze River was also blocked[63–64]. Dredging and canal contruction over the last several hundred years has increased the connections among Hongze Lake, Taihu Lake, the Yangtze River, and the East China Sea[65], but in 1954 a flood control dam was contructed at the outlet of Hongze Lake blocking upstream connectivity from the Yangtze River[66]. Taihu Lake is a geologically recent waterbody, being a large embayment of the East China Sea as recently as 1 million years ago. Gradually it became separated from the sea and is now the third largest freshwater lake in China. The hydrological connectivity among Taihu Lake, the Yangtze River, and the East China Sea has been considerably altered over the last few hundred years as a result of dam construction, canal construction, dredging, and major urbanization[67–69].

In theory, an increase of geographic distance should correlate with a gradual reduction of gene flow, resulting in genetic differentiation among populations, i.e., isolation-by-distance[70–72]. However, our results showed pairwise genetic distance values between all seven sample sites were not correlated with geographic water distance based on analyses of both microsatellites and mtDNA. Some of the patterns of genetic structure revealed is quite puzzling given that the most geographically proximate collection locations were not always the most genetically similar (e.g., Xiannv Lake and the Gan River). Some of these patterns may have been the result of anthropogenic movement of fish parasitized with glochidia of *N*. *douglasiae* for stocking and aquaculture across the region[15, 73]. Given the long history of fish stocking and aquaculture in China[15, 73], it is plausible that some of the observed pattern of genetic structure in *N*. *douglasiae* is the result of host fish stocking (moving parasiting larval mussels), but this is at best speculative until more is known about host use by *N*. *douglasiae*. It is also plausible that some of the pattern could be attributed to the movement of adult mussels for use as a human food resource, but again this is speculative.

Adult unionids disperse relatively little as adults, with long-distance dispersal being facilitated by hosts during their larval (glochidial) stage[74–75]. Given the pattern of genetic structure for *N*. *douglasiae* in the system (i.e.; similarities among Poyang Lake, Gan River, and Dongting Lake; and between Hongze Lake and Taihu Lake), it seems that at least some of the host fish species for *N*. *douglasiae* are highly vagile and capable of long distance movements through the highly hydrologically interconnected (before the active damming of rivers over the past 50 years) large river and lake system in the Yangtze River drainage, thus the maintainance of gene flow and a high degree of connectivity among habitats may be important. While there are no studies of unionids across the middle and lower Yangtze River drainage, some fish (and potential hosts for *N*. *douglasiae*) show similar patterns of genetic structure across the region[76–78]. Genetic studies of North American unionids have shown that unionids that use hosts with limited dispersal capabilities like *Epioblasma triquetra* (Rafinesque, 1820) using *Percina caprodes* [Rafinesque, 1818; Logperch] show high levels of population divergence and structure even at relatively short geographic distances[79], while unionids using highly vagile host fish capable of moving through large river systems like *Quadrula quadrula* (Rafinesque, 1820) using *Ictalurus punctatus* [Rafinesque, 1818; Channel Catfish] show lower levels of population divergence and structure and only show strong divergence and structure in populations separated by relatively high geographic distances[20, 24, 80]. Given that the host fish species of *N*. *douglasiae* are currently unknown, inferences about the dispersal abilities is impossible until experiments to determine potential host fish are conducted.

The mismatch distribution analysis and neutrality tests of the mtDNA sequence data suggest that *N*. *douglasiae* across the seven collection locations did not have a recent population expansion, and suggested that the current distribution is quite ancient. These analyses also indicate that the population dynamics of *N*. *douglasiae* are quite stable. This is not a suprising result given that *N*. *douglasiae* is a widely distributed species and often the most abundant unionid species in the region[8, 12, 61, 81]. With the Yangtze River drainage in China being among the most biodiverse regions in the world for unionid mussels[9, 61] and other aquatic organisms[12, 15, 81], this study represents an important first step for understanding the population-level diversity and structure of unionids at a regional scale.

### Genetic diversity

Higher levels of genetic diversity among populations of aquatic organisms could improve evolutionary potential for dealing with habitat change, effects of pathogen infection, and other selective forces [82–84]. The results analyses of both the mtDNA and microsatellites suggest that there is robust genetic diversity among the populations of *N*. *douglasiae* in the middle and lower Yangtze River drainage.

The analyses of the COI seqeuences showed that the haplotype diversity of *N*. *douglasiae* among the seven collection locations was high. There were more haplotypes found in *N*. *douglasiae* compared with other some rare and imperiled unionids [24, 69], but similar to other widespread species [24, 30].

The genetic diversity estimated from the microsatellite DNA results showed similar levels of genetic diversity to other unionids in the Yangtze River drainage. Mean observed heterozygocity (H_O_) expected heterozygosity (H_E_) estimated for *N*. *douglasiae* was somewhat lower than that of the widely distributed *Sinohyriopsis cumingii* (Lea, 1852; heterozygosity: 0.617–0.750)[85] and *Solenaia oleivora* (H_O_: 0.501–0.620, H_E_: 0.598–0.701)[86]. However, levels of heterozygosity calculated for *N*. *douglasiae* were somewhat higher than that estimated for *Solenaia carinata* (H_O_: 0.472, H_E_: 0.478)[32], an endemic species found only in Poyang Lake. However, these differences may simply be artifacts of the species-specific microsatellite loci used.

Virtually all *N*. *douglasiae* collection locations across the middle and lower Yangtze River drainage showed evidence of a recent bottleneck. It is unclear if these bottlenecks resulted from a founder effect due to colonization by a small founding population with low genetic diversity, or if these were the result of severe demographic reductions followed by subsequent recovery in population size. The moderate levels of genetic diversity as revealed by the polymorphic information criterion (0.25<PIC<0.5), may also be evidence for a recent genetic bottleneck.

### Conservation implications

This study represents the first analyses of the genetic structure and diversity for this widespread freshwater mussel and the first for a unionid mussel in the middle and lower Yangtze River drainage. Large-scale patterns of genetic structure occasionally differ among unionid species in the same geographic region[21, 25, 87]. Therefore, elucidating the commonalities in genetic structure and diversity among species will be necessary for making broad conservation inferences. Future research must include studies to determine dispersal capabilities of Yangtze basin unionids during all life stages[74], and studies that develop a clear understanding of the complex patterns displayed by a variety of freshwater mussel species[24]. While still poorly understood, declines in freshwater mussel populations are occurring in China[8,61]. Unionid populations in the Yangtze River region are especially vulnerable and with drastic reductions in abundance and diversity following the human disturbance and habitats fragmentation[8, 12, 61]. Currently, only two lakes (Poyang Lake and Donting Lake) remain connected with the Yangtze River. While status assessments have not been completed, it is estimated that approximately 80% of freshwater mussel species in the Yangtze River region could fall into an endangered or threatened status using IUCN criteria[12, 61]. Conservation efforts should attempt to keep individuals with similar genetic profiles together and avoid mixing of individuals from distinct genetic groups[25, 88].

In this study, *N*. *douglasiae* in the Yangtze River region showed robust genetic diversity, and significant and often high genetic differentiation (e.g., some pairwise *F*_st_>0.15) and limited gene flow among the seven collection locations. Moreover, although the historical population dynamics of *N*. *douglasiae* appear stable, loss of hydrologic connectivity among rivers and lakes in the Yangtze River drainage may lead to increased isolation of populations and possibly leading depression and population declines. Genetic structure of common species have been shown to be useful surrogates for predicting genetic structure of rare species in North American unionids[23–24]. Therefore, studies on the genetic structure and diversity of common and widespread species like *N*. *douglasiae* may assist in understanding general patterns for freshwater mussel populations in the Yangtze River drainage. At the same time, we also propose the urgent need for research on the life history of *N*. *douglasiae* and other Chinese unionids with an emphasis on characterizing habitat preferences and host-testing experiments to identify potential host fish species.

## Supporting information

S1 Fig STRUCTURE HARVESTER results to determine the most likely K value. The Evanno et al. method, which compares the ΔK between sequential K values (a) and the comparison of the mean of the estimate of the natural log of the probability of the data amongst K values, with the circle centered over the mean and the bar indicating the standard error (b).
(PDF)

S1 File Description of the development and characterization of new microsatellite loci for *N*. *douglasiae*. (DOCX)

S1 Table Characterization of the 13 microsatellite loci for *N*. *douglasiae*. (DOCX)

S2 Table Population genetic parameters in seven populations of *N*. *douglasiae*. N: number genotyped; N_A_: the number of alleles; N_E_: the effective number of alleles; H_E_: expected heterozygosity; H_O_: observed heterozygosity; *p*: significance of HWE test; F_is_:fixation index; PIC: polymorphic information content. Bold type indicates significant deviations from HWE expectations after Bonferroni correction (α = 0.0005495). Site codes as in Table 1.
(DOCX)

S3 Table Estimated null allele frequencies (Brookfield 2 method) for 13 microsatellite loci from *N*. *douglasiae* ^[^^30^^]^. Bold type indicates significant probability for the presence of null alleles.
(DOCX)

S4 Table List of all individual *Nodularia* sp. and outgroups used, collection sites, and GenBank accession codes. (DOCX)

## References

[1] Graf DL, Cummings KS. Review of the systematics and global diversity of freshwater mussel species (Bivalvia: Unionoida). Journal of Molluscan Studies. 2007; 73:291–314.

[2] He J, Zhuang Z. The freshwater bivalves of China Harxheim, Germany: ConchBooks; 2013.

[3] Vaughn CC. Ecosystem services provided by freshwater mussels. Hydrobiologia 2017; In press.

[4] Jones JW, Hallerman EM, Neves RJ. Genetic management guidelines for captive propagation of freshwater mussels (Unionoidea). Journal of Shellfish Research. 2006; 25: 527–535.

[5] Strayer DL. Freshwater Mussel Ecology: A Multifactor Approach to Distribution and Abundance. Berkeley. CA University of California Press; 2008.

[6] Barnhart MC, Haag WR, Roston WN. Adaptations to host infection and larval parasitism in Unionoida. Journal of the North American Benthological Society. 2008; 27: 370–394.

[7] Douda K, Lopes-Lima M, Hinzmann M, Machado J, Varandas S, Teixeira A, et al Biotic homogenization as a threat to native affiliate species: fish introductions dilute freshwater mussel’s resources. Diversity and Distributions. 2013; 19: 933–942.

[8] Xiong L, Ouyang S, Wu X. Fauna and standing crop of freshwater mussels in Poyang Lake, China. Chinese. Journal of Oceanology and Limnology. 2012; 30: 124–135.

[9] Zieritz A, Bogan AE, Klishko O, Kondo T, Kovitvadhi U, Kovitvadhi S, et al Diversity, biogeography and conservation status of freshwater mussels (Bivalvia: Unionida) in East and Southeast Asia. Hydrobiologia. 2017; In press.

[10] Wu X, Liang Y, Wang H, Xie Z, Ouyang S. Distribution and species diversity of freshwater mollusca of lakes along mid-lower reaches of the Yangtze river. Journal of Lake Sciences. 2000; 12: 111–118.

[11] Lopes-Lima M, Sousa R, Geist J, Aldridge DC, Araujo R, Bergengren J, et al Conservation status of freshwater mussels in Europe: state of the art and future challenges. Biological Reviews of the Cambridge Philosophical Society. 2017; 92(1): 572–607. doi: 10.1111/brv.12244 26727244 10.1111/brv.12244

[12] Shu FY, Wang HJ, Pan BZ, Liu XQ, Wang HZ. Assessment of species status of mollusca in the mid-lower Yangtze lakes. Acta Hydrobiologica Sinica. 2009; 33: 1051–1058.

[13] IUCN (International Union for Conservation of Nature). 2017. The IUCN Red List of Threatened Species. Version 2016–3. <www.iucnredlist.org>. Accessed on 22 January 2017.

[14] Zhang MH, Xu L, Xie GL, Liu YB, Liu XM, Song SC, et al Species diversity, distribution and conservation of freshwater mollusk in Poyang Lake basin. Marine Sciences. 2013; 37(8): 114–124.

[15] Fu C, Wu J, Chen J, Wu Q, Lei G. Freshwater fish biodiversity in the Yangtze River basin of China: patterns, threats and conservation. Biodiversity & Conservation. 2003; 12:1649–1685.

[16] Fraser DJ. How well can captive breeding programs conserve biodiversity? A review of Salmonids. Evolutionary Applications. 2008; 1: 535–586. doi: 10.1111/j.1752-4571.2008.00036.x 25567798 10.1111/j.1752-4571.2008.00036.x PMC3352391

[17] Liu YY, Zhang WZ, Wang YX, Wang EY. Economic Fauna of China: Freshwater Mollusks. Beijing: Science Press; 1979 pp. 116–117.

[18] Klishko OK, Lopes-Lima M, Froufe E, Bogan AE, Abakumova VY. Unravelling the systematics of *Nodularia* (Bivalvia, Unionidae) species from eastern Russia. Systematics and Biodiversity. 2017; 1–15.

[19] Elderkin CL, Christian AD, Vaughn CC, Metcalfe-Smith JL, Berg DJ. Population genetics of the freshwater mussel, *Amblema plicata* (Say 1817) (Bivalvia: Unionidae): Evidence of high dispersal and post-glacial colonization. Conservation Genetics. 2007; 8: 355–372.

[20] Mathias PT, Hoffman JR, Wilson CC, Zanatta DT. Signature of postglacial colonization on contemporary genetic structure and diversity of *Quadrula quadrula* (Bivalvia: Unionidae). Hydrobiologia. 2017; In press.

[21] Elderkin CL, Christian AD, Metcalfe-Smith JL, Berg DJ. Population genetics and phylogeography of freshwater mussels in North America, *Ellipto dilatata* and *Actinonaias ligamentina* (Bivalvia: Unionidae). Molecular Ecology. 2008; 17: 2149–2163. doi: 10.1111/j.1365-294X.2008.03745.x 18410287 10.1111/j.1365-294X.2008.03745.x

[22] Haag WR. Williams JD. Biodiversity on the brink: an assessment of conservation strategies for North American freshwater mussels. Hydrobiologia. 2014; 735: 45–60.

[23] Berg DJ, Cantonwine EG, Hoeh WR, Guttman SI. Genetic structure of *Quadrula quadrula* (Bivalvia: Unionidae): little variation across large distances. Journal of Shellfish Research. 1998; 17: 1365–1373.

[24] Galbraith HS, Zanatta DT, Wilson CC. Comparative analysis of riverscape genetic structure in rare, threatened and common freshwater mussels. Conservation Genetics. 2015; 16: 845–857.

[25] Zhang Q, Li L, Wang YG, Werner AD, Xin P, Jiang T, et al Has the Three-Gorges Dam made the Poyang Lake wetlands wetter and drier? Geophysics Research Letters. 2012; 39(20): L20402.1–L20402.7.

[26] Gissi C, Iannelli F, Pesole G. Evolution of the mitochondrial genome of Metazoa as exemplified by comparison of congeneric species. Heredity. 2008; 101(4): 301–320. doi: 10.1038/hdy.2008.62 18612321 10.1038/hdy.2008.62

[27] Doucet-Beaupré H, Breton S, Chapman EG, Blier PU, Bogan AE et al Mitochondrial phylogenomics of the Bivalvia (Mollusca): searching for the origin and mitogenomic correlates of doubly uniparental inheritance of mtDNA. BMC Evol Biol. 2010; 10(1): 50–67. 20167078 10.1186/1471-2148-10-50 PMC2834691

[28] Stöger I, Schrödl M. Mitogenomics does not resolve deep molluscan relationships (yet?). Molecular Phylogenetics and Evolution. 2013; 69 (2): 376–392. doi: 10.1016/j.ympev.2012.11.017 23228545 10.1016/j.ympev.2012.11.017

[29] Collier KJ, Probert PK, Jeffries M. Conservation of aquatic invertebrates: concerns, challenges and conundrums. Aquatic Conservation: Marine and Freshwater Ecosystems. 2016; 26: 817–837.

[30] Zanatta DT, Murphy RW. The phylogeographical and management implications of genetic population structure in the imperiled snuffbox mussel, *Epioblasma triquetra* (Bivalvia: Unionidae). Biological Journal of the Linnean Society. 2008; 93: 371–384.

[31] Geist J, Geismar J, Kuehn R. Isolation and characterization of the first microsatellite markers for the endangered swan mussel *Anodonta cygnea* L. (Bivalvia: Unionoidea). Conservation Genetics. 2010; 11: 1103–1106.

[32] Sun TT, Liu X, Zhou C, Ding H, Yang W, Zanatta DT, et al Microsatellite analysis of genetic diversity and genetic structure of the Chinese freshwater mussel *Solenaia carinata* (Bivalvia: Unionidae). Aquatic Conservation: Marine and Freshwater Ecosystems. 2017; In press.

[33] DeWoody JA, Schupp J, Kenefic L, Busch J, Murfitt L, Keim P. Universal method for producing ROX-labeled size standards suitable for automated genotyping. Biotechniques. 2004; 37:348–353. 15470886 10.2144/04373BM02

[34] Thompson JD, Gibson TJ, Plewinak F. The Clustal2X windows interface: flexible strategies ed for multiple sequences alignment aided by quality analysis, tools. Nucleic Acids Research. 1997; 25 (24): 4876–4882. 9396791 10.1093/nar/25.24.4876 PMC147148

[35] Librado P, Rozas J. DnaSP v5: a software for comprehensive analysis of DNA polymorphism data. Bioinformatics, 2009; 25(11):1451 doi: 10.1093/bioinformatics/btp187 19346325 10.1093/bioinformatics/btp187

[36] Ronquist F, Teslenko M, van der Mark P, Ayres DL, Darling AS, et al MrBayes 3.2: Efficient Bayesian phylogenetic inference and model choice across a large model space. Systematic Biology. 2012; 61: 539–542. doi: 10.1093/sysbio/sys029 22357727 10.1093/sysbio/sys029 PMC3329765

[37] Nylander JAA. MrModeltest v2. Program distributed by the author Uppsala: Evolutionary Biology Centre, Uppsala University; 2004.

[38] Clement M, Snell Q, Walke P, Posada D, Crandall, K. TCS: estimating gene genealogies. Proceedings of the 16th International Parallel Distributions Process Symposium. 2002; 2:184.

[39] Leigh JW, Bryant D. PopART: Full-feature software for haplotype network construction. Methods in Ecology and Evolution. 2015; 6(9):1110–1116.

[40] Excoffier L, Lischer HE. Arlequin suite ver 3.5: a new series of programs to perform population genetics analyses under Linux and Windows. Molecular Ecology Resources. 2010; 10(3): 564–567. doi: 10.1111/j.1755-0998.2010.02847.x 21565059 10.1111/j.1755-0998.2010.02847.x

[41] Jensen JL, Bohonak AJ, Kelley ST. Isolation by distance, web service. BMC Genetics. 2005; 6: 13 v.3.23 http://ibdws.sdsu.edu/ doi: 10.1186/1471-2156-6-13 15760479 10.1186/1471-2156-6-13 PMC1079815

[42] Drummond AJ, Rambaut A, Shapiro B, Pybus OG. Bayesian coalescent inference of past population dynamics from molecular sequences. Molecular Biology and Evolution. 2005; 22: 1185–1192. doi: 10.1093/molbev/msi103 15703244 10.1093/molbev/msi103

[43] Drummond AJ, Rambaut A. BEAST: Bayesian evolutionary analysis by sampling trees. BMC Evolutionary Biology. 2007; 7: 214 doi: 10.1186/1471-2148-7-214 17996036 10.1186/1471-2148-7-214 PMC2247476

[44] Rambaut A, Drummond AJ. Tracer v1.5. http://beast.bio.ed.ac.uk/Tracer.2007.

[45] Yeh FC, Yang R, Boyle TJ, Ye Z, Xian JM. POPGENE32, Microsoft Windows-based free-ware for population genetic analysis, version 1.32 Canada: Molecular Biology and Biotechnology, University of Alberta, Edmonton; 2000.

[46] Kalinowski ST, Taper ML, Marshall TC. Revising how the computer program CERVUS accommodates genotyping error increases success in paternity assignment. Molecular Ecology. 2007; 16: 1099–1106. doi: 10.1111/j.1365-294X.2007.03089.x 17305863 10.1111/j.1365-294X.2007.03089.x

[47] van Oosterhout C, Hutchinson WF, Wills DPM., Shipley P. MICRO-CHECKER: Software for identifying and correcting genotyping errors in microsatellite data. Molecular Ecology Notes. 2004; 4: 535–538.

[48] Piry S, Luikart G, Cornuet JM. BOTTLENECK: a computer program for detecting recent reductions in the effective population size using allele frequency data. Journal of Heredity. 1999; 90: 502–503.

[49] Pritchard JK, Stephens M, Donnelly P. Inference of population structure using multilocus genotype data. Genetics. 2000; 155: 945–949. 10835412 10.1093/genetics/155.2.945 PMC1461096

[50] Evanno G, Regnaut S, Goudet J. Detecting the number of clusters of individuals using the software structure: a simulation study. Molecular Ecology, 2005; 14: 2611–2620. doi: 10.1111/j.1365-294X.2005.02553.x 15969739 10.1111/j.1365-294X.2005.02553.x

[51] Earl D, Von Holdt B. STRUCTURE HARVESTER: A website and program for visualizing STRUCTURE output and implementing the Evanno method. Conservation Genetics Resources, 2012; 4: 359–361.

[52] Peakall R, Smouse PE. GenAlEx 6.5: Genetic analysis in Excel. Population genetic software for teaching and research-an update. Bioinformatics. 2012; 28: 2537–2539. doi: 10.1093/bioinformatics/bts460 22820204 10.1093/bioinformatics/bts460 PMC3463245

[53] Nei M. Genetic distance between populations. American Naturalist. 1972; 106: 283–292.

[54] Excoffier L, Smouse PE, Quattro JM. Analysis of molecular variance inferred from metric distances among DNA haplotypes: application to human mitochondrial DNA restriction data. Genetics. 1992; 131: 479–491. 1644282 10.1093/genetics/131.2.479 PMC1205020

[55] Jost L. G_ST_ and its relatives do not measure differentiation. Molecular Ecology. 2008; 17: 4015–4026. 19238703 10.1111/j.1365-294x.2008.03887.x

[56] Dakin EE, Avise JC. Microsatellite null alleles in parentage analysis. Heredity. 2004; 93:504–509. doi: 10.1038/sj.hdy.6800545 15292911 10.1038/sj.hdy.6800545

[57] Carlsson J. Effects of microsatellite null alleles on assignment testing. Journal of Heredity. 2008; 99:616–623. doi: 10.1093/jhered/esn048 18535000 10.1093/jhered/esn048

[58] Astanei IE, Gosling JW, Powell E. Genetic variability and phylogeography of the invasive zebra mussel, *Dreissena polymorpha* (Pallas). Molecular Ecology. 2005; 14: 1655–1666. doi: 10.1111/j.1365-294X.2005.02530.x 15836640 10.1111/j.1365-294X.2005.02530.x

[59] Li G, Hubert S, Bucklin K, Ribes V, Hedgecock D. Characterization of 79 microsatellite DNA markers in the Pacific oyster, *Crassostrea gigas*. Molecular Ecology Notes. 2003; 3: 228–232.

[60] Wright S. The interpretation of population structure by F-statistics with special regard to systems of mating. Evolution, 1965; 19: 395–420.

[61] Wu XP. Studies on Freshwater Mollusca in Mid reaches of Changjiang River. Ph. D. Thesis. Wuhan: Institute of Hydrobiology, Chinese Academy of Sciences; 1998.

[62] Li WH, Zhang M, Meng JS, Ao XF, Hu XY, Ouyang S, et al Community structure of macrozoobenthos in Xiannv Lake basin and assessment of its water. Resources and Environment in the Yangtze Basin. 2016; 25: 1218–1227.

[63] Zhang QH, Dong XH, Yang XD. Environmental evolution of Lake Liangzi and its driving factors over the past 100 years, Hubei Province. Journal of Lake Sciences. 2016; 28(3):545–553.

[64] Li ZH, Sun DZ. Study on ecological environmental protection in Liangzi Lake Beijing: Science Press; 2009.

[65] Wang Q, Chen JY. Formation and evolution of Hongze Lake and the Huaihe River mouth along the lake. Journal of Lake Sciences. 1999; 11(3): 237–244.

[66] Han SQ. Research on the processes and backgrounds of the changes of the Honze Lake during Historical period. Journal of Chinese Historical Geography. 1998; 2: 61–76.

[67] Liu JL. The origin and evolution of Taihu Lake: a 11000 year journal. Acta Palaeontologica Sinica. 1996; 35(2): 129–135.

[68] Hong XQ. Origin and evolution of the Thaihu Lake. Marine geology and quaternary geology. 1991; 11(4): 87–99.

[69] Jing CY. Formation and evolution of Taihu Lake. Scientia geographica sinica. 1989; 9(4): 378–385.

[70] Barton NH, Kelleher J, Etheridge AM. A new model for extinction and recolonization in two dimensions: quantifying phylogeography. Evolution. 2010; 64: 2701–2715. doi: 10.1111/j.1558-5646.2010.01019.x 20408876 10.1111/j.1558-5646.2010.01019.x

[71] Hurtrez-Boussès S, Hurtrez JE, Turpin H, Durand C, Durand P, De Meeus T, et al Hydrographic network structure and population genetic differentiation in a vector of fasciolosis, *Galba truncatula*. Infection Genetics and Evolution. 2010; 10: 178–183. 10.1016/j.meegid.2010.01.005 20085826

[72] Husemann M, Ray JW, King RS, Hooser EA, Danley PD. Comparative biogeography reveals differences in population genetic structure of five species of stream fishes. Biological Journal of the Linnean Society. 2012; 107: 867–885.

[73] Zhu B, Zheng HT, Que YF, Chang JB. Fish stocking program in the Yangtze River. Chinese Fisheries Economics. 2009; 27:74–87.

[74] Ferguson CD, Blum MJ, Raymer ML, Eackles MS, Krane DE. Population structure, multiple paternity, and long-distance transport of spermatozoa in the freshwater mussel *Lampsilis cardium* (Bivalvia:Unionidae). Freshwater Science. 2013; 32:267–282.

[75] Haag WR. North American Freshwater Mussels: Natural History, Ecology and Conservation. Cambridge UK, Cambridge University Press; 2012.

[76] Liao X, Yu X, Tong J. Genetic diversity of common carp from two largest Chinese lakes and the Yangtze River revealed by microsatellite markers. Hydrobiologia. 2006; 568:445–453.

[77] Wang C, Yu X, Tong J. Microsatellite diversity and population genetic structure of redfin culter (*Culter erythropterus*) in fragmented lakes of the Yangtze River. Hydrobiologia. 2007; 586:321–329.

[78] Abbas K, Zhou X, Li Y, Gao Z, Wang W. Microsatellite diversity and population genetic structure of yellowcheek, *Elopichthys bambusa* (Cyprinidae) in the Yangtze River. Biochemical Systematics and Ecology. 2010; 38:806–812

[79] Zanatta DT, Wilson CC. Testing congruency of geographic and genetic population structure for a freshwater mussel (Bivalvia: Unionoida) and its host fish. Biological Journal of the Linnean Society. 2011; 102: 669–685.

[80] Paterson WL, Griffith TA, Burlakova LE, Krebs RW, Zanatta DT. An evaluation of the genetic structure of mapleleaf mussels (*Quadrula quadrula*) in the Lake Erie watershed. Journal of Great Lakes Research. 2015; 41: 1123–1130.

[81] Zhang X, Lin ZT. Morphology of freshwater mussel and common species in China. Bulletin of Biology. 1959; (5):14–22.

[82] Freeland JL, Petersen SD, Kirk H. Molecular Ecology, (2nd ed.). Oxford, UK: Wiley-Blackwell 2011.

[83] Liu XH, Yao YG. Characterization of 12 polymorphic microsatellite markers in the Chinese tree shrew (*Tupaia belangeri chinensis*). Zoological Research. 2013; 34: E62–E68. doi: 10.3724/SP.J.1141.2013.E02E62 23572368 10.3724/SP.J.1141.2013.E02E62

[84] Wu SZ, Guan YY, Huang XD, He MX. Development of 25 novel microsatellite loci and genetic variation analysis in breeding populations of the pearl oyster, *Pinctada fucata*. Journal of the World Aquaculture Society. 2013; 44: 600–609.

[85] Luo YM. Genetic diversity of four *Hyriopsis cumingii* (Bivalvia: Unionidae) populations by SSR analysis. Hubei: Huazhong Agricultural University; 2006.

[86] Xu Y. Isolation of microsatellite markers and population genetic diversity analysis in *Solenaia oleivora* Wuhan: Huazhong Agricultural University; 2014.

[87] Scott MW, Begley MT, Krebs RA, Zanatta DT. Mitochondrial DNA variation in the Eastern Pondmussel, *Ligumia nasuta* (Bivalvia: Unionoida), in the Great Lakes region. Walkerana. 2014; 17: 60–67.

[88] Hoftyzer E, Ackerman JD, Morris TJ, Mackie GL. Genetic and environmental implications of reintroducing laboratory-raised unionid mussels into the wild. Canadian Journal of Fisheries and Aquatic Sciences. 2008; 65: 1217–1229.

