# Supplementary material for: Genetic structure and diversity of Nodularia douglasiae (Bivalvia: Unionida) from the middle and lower Yangtze River drainage
Source: PLoS One. 2017 Dec 20;12(12):e0189737. doi: 10.1371/journal.pone.0189737 (PMC5738091; doi:10.1371/journal.pone.0189737)
Supplement: S1 Fig — The Evanno et al. method, which compares the ΔK between sequential K values (a) and the comparison of the mean of the estimate of the natural log of the probability of the data amongst K values, with the circle centered over the mean and the bar indicating the standard error (b). (PDF) [file pone.0189737.s001.pdf]

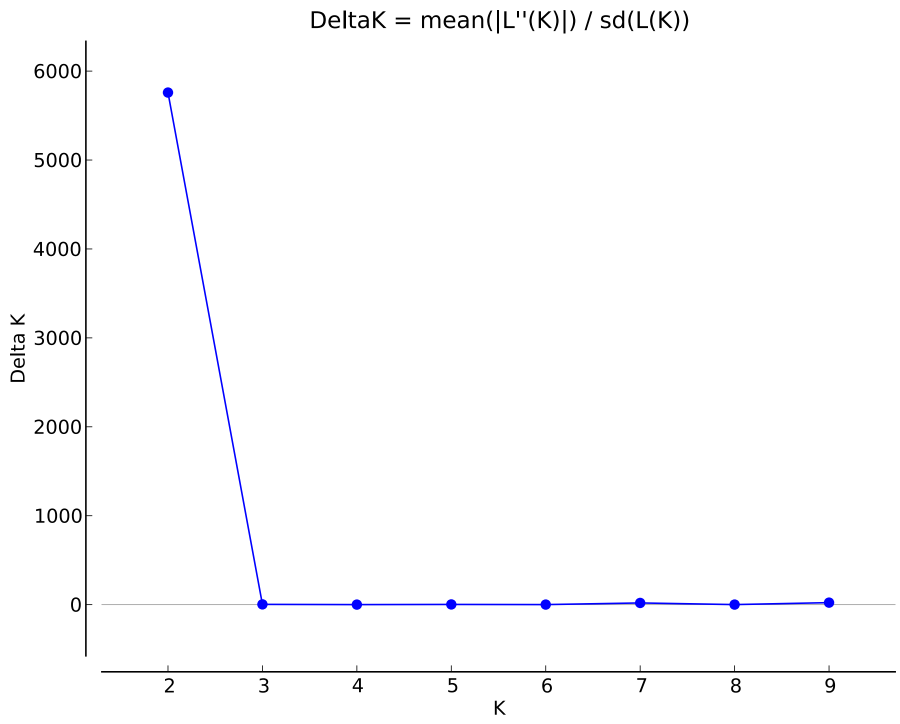

a)

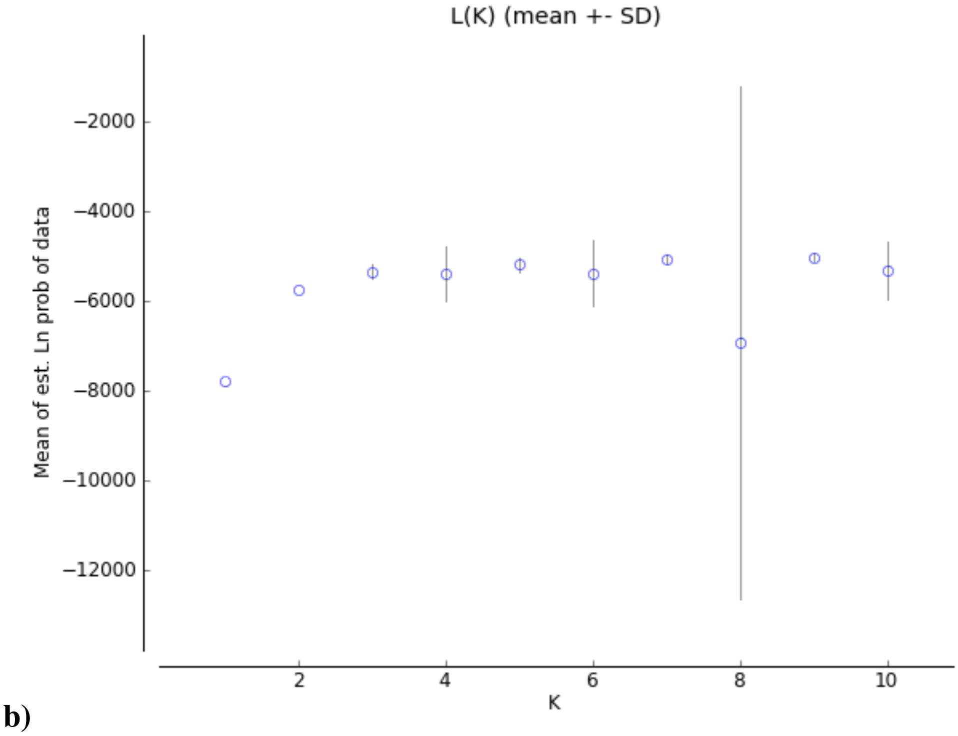

b)

S1 Fig. STRUCTURE HARVESTER results to determine the most likely *K* value. The Evanno et al. method, which compares the ΔK between sequential *K* values (a) and the comparison of the mean of the estimate of the natural log of the probability of the data amongst *K* values, with the circle centered over the mean and the bar indicating the standard error (b).
