# Supplementary material for: Genetic structure and diversity of Nodularia douglasiae (Bivalvia: Unionida) from the middle and lower Yangtze River drainage
Source: PLoS One. 2017 Dec 20;12(12):e0189737. doi: 10.1371/journal.pone.0189737 (PMC5738091; doi:10.1371/journal.pone.0189737)
Supplement: S1 File — (DOCX) [file pone.0189737.s002.docx]

**Supporting Infomation**

**S1 File. Description of the development and characterization of new microsatellite loci for *N. douglasiae***

RNA was extracted from mantle tissue of *N. douglasiae* using the Eastep Universal RNA Extraction Kit (Promega) from two *N. douglasiae* individuals from Poyang Lake (Jiangxi Province, China). Concentration and quality of RNA were estimated using a Nanodrop 2000. A total of over 92 million unigenes were obtained from which 16731 microsatellite sequences were detected using MISA (MIcroSAtellite identification tool; http://pgrc.ipk-gatersleben.de/misa/). After successful transcriptome sequencing, 100 pairs of primers were designed using PRIMER 3.0 ONLINE^[97]^.

Genomic DNA was extracted from mantle tissue of 40 *U. douglasiae* collected from Poyang Lake using the TINAamp Marine Animals DNA Kit. Concentration and quality of DNA were estimated using a Nanodrop 2000 (Thermo Scientific) and agarose gel electrophoresis. Each primer pair was screened for reliable amplification using 40 individuals of *N. douglasiae*. The PCR reactions, amplication conditions, and scoring of alleles used for screening the newly developed loci are as described in the methods. Genbank Accession numbers for the newly developed microsatellite markers are KX673748-KX673761. Twelve of the 14 loci developed and characterized for *N. douglasiae* were used in this study
