# Supplementary material for: Genetic structure and diversity of Nodularia douglasiae (Bivalvia: Unionida) from the middle and lower Yangtze River drainage
Source: PLoS One. 2017 Dec 20;12(12):e0189737. doi: 10.1371/journal.pone.0189737 (PMC5738091; doi:10.1371/journal.pone.0189737)
Supplement: S1 Table — (DOCX) [file pone.0189737.s003.docx]

**S1 Table.** Characterization of the 13 microsatellite loci for *N. douglasiae*.

| Locus | Primer sequence (5′– 3′) | GenBank Accession | Annealing Temperature (℃) | Repeat motif | Size (bp) |
| --- | --- | --- | --- | --- | --- |
| Udo1 | F: ACGGGACATAGGACCATACCA  R: TGCACTTTAGCTGTCAAGAGAC | KX673748 | 56.3 | (TA)_22_ | 192-303 |
| Udo2 | F: ACGGGCGGCATTCTTTAGTT  R: ACCAGAACGTGACTTTGGCT | KX673749 | 56.3 | (CAT)_12_ | 156-290 |
| Udo3 | F: CTTGTCACTGCCTTCCCTGA  R: GGGATCTGCAAGCACACCAT | KX673750 | 57.0 | (GA)_43_ | 154-333 |
| Udo4 | F: TGCCATGGTGAACACTTCTGT  R: ACCAAATTTGCATGCTCAGGT | KX673751 | 56.3 | (GT)_14_ | 158-313 |
| Udo5 | F: TTTGAATGCAGGGGAGAGGG  R: TGAGACTGATATGAGTGCAGGA | KX673752 | 51.7 | (TT)_11_(AA)_15_ | 176-301 |
| Udo6 | F: AGTGCTCCATCAACAAAGTGA  R: GCCACATCAGTTGAGGTTTCC | KX673753 | 52.5 | (TC)_18_ | 156-230 |
| Udo7 | F: CAGAAGGTGGCTGGGAAAGT  R: TGCACACACACTGGCATACA | KX673754 | 52.5 | (GT)_25_ | 151-323 |
| Udo8 | F:TGAATGAGCTTGAAATTTAATGTGAGT  R: TGCTCGCTAACCATCTCACC | KX673755 | 61.4 | (TGA)_11_ | 155-292 |
| Udo9 | F: ATTCCAGCACAGCAGACTCC  R: GCCCCAGCCTTCATGATTCT | KX673756 | 64.6 | (CAG)_18_ | 151-433 |
| Udo10 | F: TGGCACTCCTGAAAGATAGGT  R: GGTAGGCATATCTGTACACTGCA | KX673757 | 51.7 | (AT)_25_ | 206-386 |
| Udo11 | F: TCCGAACTGTGTTGATGGCT  R: CCTGATTCTGTGGTTCTGGCT | KX673758 | 56.3 | (TA)_36_ | 173-242 |
| Udo14 | F: TTGCCATCCTTGAGCCACTT  R: ACCCAGTGACCTCATCTCCA | KX673761 | 63.4 | (CA)_53_ | 718-968 |
| Scasst17 | F: CCCAGAGCACACACACAAAC  R: AGCACCAACACATCTTGCCT | KU572461 | 55.9 | (AC)_n_ | 117-217 |
