# Supplementary material for: Genetic structure and diversity of Nodularia douglasiae (Bivalvia: Unionida) from the middle and lower Yangtze River drainage
Source: PLoS One. 2017 Dec 20;12(12):e0189737. doi: 10.1371/journal.pone.0189737 (PMC5738091; doi:10.1371/journal.pone.0189737)
Supplement: S2 Table — N: number genotyped; NA: the number of alleles; NE: the effective number of alleles; HE: expected heterozygosity; HO: observed heterozygosity; p: significance of HWE test; Fis:fixation index; PIC: polymorphic information content. Bold type indicates significant deviations from HWE expectations after Bonferroni correction (α = 0.0005495). Site codes as in Table 1. (DOCX) [file pone.0189737.s004.docx]

**S2 Table.** Population genetic parameters in seven populations of *N. douglasiae*. N: number genotyped; N_A_: the number of alleles; N_E_: the effective number of alleles; H_E_: expected heterozygosity; H_O_: observed heterozygosity; *p*: significance of HWE test; F_is_:fixation index; PIC: polymorphic information content. Bold type indicates significant deviations from HWE expectations after Bonferroni correction (α = 0.0005495). Site codes as in Table 1.

| loci | LZ | DT | PY | GJ | XN | HZ | TH |
| --- | --- | --- | --- | --- | --- | --- | --- |
| **Udo1** |  |  |  |  |  |  |  |
| N | 10 | 34 | 32 | 32 | 30 | 30 | 30 |
| N_A_ | 2 | 4 | 3 | 2 | 2 | 4 | 2 |
| N_E_ | 1.1 | 1.9 | 1.8 | 1.4 | 1.4 | 1.6 | 1.3 |
| H_E_ | 0.10 | 0.51 | 0.46 | 0.41 | 0.27 | 0.38 | 0.21 |
| H_O_ | 0.10 | 0.32 | 0.33 | 0.33 | 0.24 | 0.47 | 0.23 |
| *p* | 1.000 | 0.321 | **<0.001** | 0.002 | 0.609 | 0.673 | 0.372 |
| F_is_ | -0.0526 | 0.4528 | 0.7696 | 0.8552 | 0.275 | -0.1661 | -0.1321 |
| PIC | 0.39 | 0.393 | 0.385 | 0.377 | 0.344 | 0.289 | 0.384 |
| **Udo2** |  |  |  |  |  |  |  |
| N | 10 | 34 | 32 | 32 | 30 | 30 | 30 |
| N_A_ | 2 | 2 | 2 | 2 | 2 | 3 | 3 |
| N_E_ | 1.5 | 1.2 | 1.4 | 1.4 | 1.2 | 1.2 | 1.2 |
| H_E_ | 0.34 | 0.36 | 0.29 | 0.27 | 0.16 | 0.23 | 0.19 |
| H_O_ | 0.40 | 0.48 | 0.34 | 0.31 | 0.17 | 0.31 | 0.2 |
| *p* | 0.383 | 0.486 | 0.148 | 0.195 | 0.539 | 0.851 | 0.906 |
| F_is_ | -0.25 | -0.0968 | -0.1636 | -0.2308 | -0.0943 | -0.0526 | -0.0169 |
| PIC | 0.268 | 0.347 | 0.244 | 0.258 | 0.345 | 0.29 | 0.33 |
| **Udo3** |  |  |  |  |  |  |  |
| N | 10 | 34 | 32 | 32 | 30 | 30 | 30 |
| N_A_ | 5 | 8 | 5 | 9 | 3 | 7 | 8 |
| N_E_ | 3.8 | 1.4 | 1.3 | 2.1 | 1.9 | 1.7 | 1.8 |
| H_E_ | 0.77 | 0.40 | 0.44 | 0.51 | 0.48 | 0.40 | 0.43 |
| H_O_ | 0.70 | 0.32 | 0.39 | 0.56 | 0.72 | 0.47 | 0.50 |
| *p* | 0.054 | 1.000 | 0.778 | 1.000 | 0.007 | 0.999 | 1.000 |
| F_is_ | 0.0476 | 0.1577 | 0.1847 | -0.1301 | -0.5282 | -0.2245 | -0.1749 |
| PIC | 0.689 | 0.386 | 0.223 | 0.504 | 0.376 | 0.438 | 0.411 |
| **Udo4** |  |  |  |  |  |  |  |
| N | 10 | 34 | 32 | 32 | 30 | 30 | 30 |
| N_A_ | 3 | 6 | 6 | 4 | 3 | 3 | 3 |
| N_E_ | 1.5 | 1.3 | 1.6 | 1.1 | 1.3 | 1.1 | 1.1 |
| H_E_ | 0.35 | 0.52 | 0.35 | 0.39 | 0.25 | 0.34 | 0.35 |
| H_O_ | 0.20 | 0.48 | 0.32 | 0.36 | 0.14 | 0.33 | 0.33 |
| *p* | 0.305 | 0.709 | 0.82 | 0.113 | 0.119 | 0.017 | 0.017 |
| F_is_ | 0.403 | 0.153 | 0.3645 | 0.3118 | 0.43 | 0.6532 | 0.4737 |
| PIC | 0.302 | 0.312 | 0.317 | 0.389 | 0.319 | 0.29 | 0.322 |
| **Udo5** |  |  |  |  |  |  |  |
| N | 10 | 34 | 32 | 32 | 30 | 30 | 30 |
| N_A_ | 3 | 7 | 7 | 5 | 3 | 4 | 5 |
| N_E_ | 1.7 | 4 | 2.9 | 2.8 | 2.2 | 2.1 | 2 |
| H_E_ | 0.42 | 0.76 | 0.67 | 0.66 | 0.56 | 0.54 | 0.51 |
| H_O_ | 0.30 | 0.76 | 0.75 | 0.50 | 0.90 | 0.30 | 0.40 |
| *p* | 0.585 | 0.027 | 0.397 | **<0.001** | 0.001 | **<0.001** | 0.42 |
| F_is_ | 0.2405 | -0.0231 | -0.1361 | 0.2272 | -0.6409 | 0.8753 | 0.2044 |
| PIC | 0.346 | 0.712 | 0.585 | 0.596 | 0.443 | 0.448 | 0.455 |
| **Udo6** |  |  |  |  |  |  |  |
| N | 10 | 34 | 32 | 32 | 30 | 30 | 30 |
| N_A_ | 4 | 2 | 2 | 2 | 2 | 3 | 2 |
| N_E_ | 1.5 | 1.3 | 1.2 | 1.1 | 1.1 | 1.9 | 1.9 |
| H_E_ | 0.36 | 0.41 | 0.35 | 0.39 | 0.2 | 0.5 | 0.47 |
| H_O_ | 0.20 | 0.38 | 0.36 | 0.39 | 0.20 | 0.77 | 0.73 |
| *p* | 0.046 | 0.373 | 0.561 | 0.75 | 0.74 | 0.002 | **<0.001** |
| F_is_ | 0.4203 | 0.15 | -0.0847 | -0.0492 | -0.0545 | -0.7666 | -0.8182 |
| PIC | 0.325 | 0.386 | 0.333 | 0.385 | 0.39 | 0.395 | 0.372 |
| **Udo7** |  |  |  |  |  |  |  |
| N | 10 | 34 | 32 | 32 | 30 | 30 | 30 |
| N_A_ | 6 | 7 | 6 | 7 | 4 | 7 | 7 |
| N_E_ | 3.9 | 4.8 | 3 | 4 | 2.1 | 3.7 | 2.8 |
| H_E_ | 0.78 | 0.8 | 0.68 | 0.76 | 0.53 | 0.74 | 0.65 |
| H_O_ | 1.00 | 0.56 | 0.56 | 0.56 | 0.41 | 0.90 | 0.90 |
| *p* | 0.087 | 0.018 | 0.087 | 0.013 | 0.036 | 0.023 | 0.255 |
| F_is_ | -0.3423 | 0.2948 | 0.1567 | 0.2524 | 0.1354 | -0.1199 | -0.2121 |
| PIC | 0.709 | 0.761 | 0.621 | 0.711 | 0.415 | 0.746 | 0.689 |
| **Udo8** |  |  |  |  |  |  |  |
| N | 10 | 34 | 32 | 32 | 30 | 30 | 30 |
| N_A_ | 3 | 2 | 3 | 3 | 2 | 2 | 2 |
| N_E_ | 1.5 | 1.4 | 1.3 | 1.3 | 1 | 1.1 | 1.1 |
| H_E_ | 0.36 | 0.31 | 0.26 | 0.41 | 0.33 | 0.1 | 0.33 |
| H_O_ | 0.20 | 0.26 | 0.22 | 0.39 | 0.33 | 0.30 | 0.34 |
| *p* | 0.046 | 0.37 | 0.817 | 0.001 | 1.000 | 0.745 | 0.643 |
| F_is_ | 0.4118 | 0.1441 | 0.1267 | 0.3621 | -0.0175 | -0.7345 | -0.0714 |
| PIC | 0.314 | 0.261 | 0.322 | 0.341 | 0.333 | 0.262 | 0.316 |
| **Udo9** |  |  |  |  |  |  |  |
| N | 10 | 34 | 32 | 32 | 30 | 30 | 30 |
| N_A_ | 3 | 6 | 5 | 8 | 4 | 5 | 4 |
| N_E_ | 1.7 | 1.4 | 1.2 | 1.3 | 2.1 | 1.6 | 1.6 |
| H_E_ | 0.42 | 0.27 | 0.35 | 0.26 | 0.53 | 0.36 | 0.38 |
| H_O_ | 0.50 | 0.22 | 0.32 | 0.22 | 0.90 | 0.43 | 0.46 |
| *p* | 0.714 | 0.003 | 0.666 | 0.998 | **<0.001** | 0.971 | 0.673 |
| F_is_ | -0.2658 | 0.5606 | 0.1579 | 0.1499 | -0.7117 | -0.125 | -0.2131 |
| PIC | 0.346 | 0.26 | 0.345 | 0.25 | 0.418 | 0.251 | 0.321 |
| **Udo10** |  |  |  |  |  |  |  |
| N | 10 | 34 | 32 | 32 | 30 | 30 | 30 |
| N_A_ | 2 | 5 | 6 | 3 | 3 | 4 | 6 |
| N_E_ | 1.6 | 1.5 | 1.4 | 1.1 | 1.5 | 2.5 | 2.4 |
| H_E_ | 0.36 | 0.32 | 0.29 | 0.32 | 0.35 | 0.6 | 0.59 |
| H_O_ | 0.30 | 0.42 | 0.23 | 0.36 | 0.24 | 0.23 | 0.43 |
| *p* | 0.43 | **<0.001** | 0.130 | 0.115 | 0.020 | **<0.001** | 0.072 |
| F_is_ | 0.2 | 0.6217 | 0.6454 | 0.4667 | 0.2988 | 0.5357 | -0.0049 |
| PIC | 0.304 | 0.296 | 0.264 | 0.31 | 0.31 | 0.595 | 0.508 |
| **Udo11** |  |  |  |  |  |  |  |
| N | 10 | 34 | 32 | 32 | 30 | 30 | 30 |
| N_A_ | 3 | 7 | 5 | 7 | 5 | 7 | 3 |
| N_E_ | 2.9 | 3.4 | 3.4 | 3.2 | 1.9 | 4.4 | 2.3 |
| H_E_ | 0.69 | 0.72 | 0.72 | 0.7 | 0.46 | 0.79 | 0.58 |
| H_O_ | 0.50 | 0.59 | 0.72 | 0.66 | 0.55 | 0.77 | 0.53 |
| *p* | 0.594 | 0.756 | 0.107 | 0.117 | 0.826 | 0.949 | 0.366 |
| F_is_ | 0.2366 | 0.196 | -0.0173 | 0.0461 | -0.1762 | 0.0625 | 0.0634 |
| PIC | 0.58 | 0.661 | 0.648 | 0.647 | 0.437 | 0.748 | 0.476 |
| **Udo14** |  |  |  |  |  |  |  |
| N | 10 | 34 | 32 | 32 | 30 | 30 | 30 |
| N_A_ | 3 | 11 | 11 | 17 | 17 | 8 | 14 |
| N_E_ | 3.7 | 6.5 | 7.7 | 8.3 | 7.5 | 4.7 | 6.9 |
| H_E_ | 0.66 | 0.85 | 0.88 | 0.89 | 0.88 | 0.8 | 0.87 |
| H_O_ | 0.50 | 0.47 | 0.53 | 0.50 | 0.48 | 0.50 | 0.50 |
| *p* | 0.001 | **<0.001** | 0.032 | 0.341 | 0.611 | **<0.001** | 0.008 |
| F_is_ | 0.2 | 0.4429 | 0.3535 | 0.4202 | 0.429 | 0.3333 | 0.3988 |
| PIC | 0.554 | 0.828 | 0.856 | 0.853 | 0.835 | 0.717 | 0.818 |
| **Scastt17** |  |  |  |  |  |  |  |
| N | 10 | 34 | 32 | 32 | 30 | 30 | 30 |
| N_A_ | 3 | 5 | 5 | 5 | 5 | 5 | 4 |
| N_E_ | 2.5 | 2.9 | 2.7 | 2.7 | 3.8 | 3.7 | 2.8 |
| H_E_ | 0.64 | 0.67 | 0.63 | 0.64 | 0.75 | 0.74 | 0.65 |
| H_O_ | 1.00 | 1.00 | 0.91 | 0.94 | 1.00 | 1.00 | 1.00 |
| *p* | 0.005 | 0.061 | 0.002 | 0.052 | **<0.001** | 0.055 | **<0.001** |
| F_is_ | -0.6529 | -0.5281 | -0.4421 | -0.4942 | -0.3532 | -0.3772 | -0.5557 |
| PIC | 0.526 | 0.602 | 0.582 | 0.573 | 0.699 | 0.683 | 0.583 |
