# Supplementary material for: Genetic structure and diversity of Nodularia douglasiae (Bivalvia: Unionida) from the middle and lower Yangtze River drainage
Source: PLoS One. 2017 Dec 20;12(12):e0189737. doi: 10.1371/journal.pone.0189737 (PMC5738091; doi:10.1371/journal.pone.0189737)
Supplement: S3 Table — Bold type indicates significant probability for the presence of null alleles. (DOCX) [file pone.0189737.s005.docx]

**S3 Table.** Estimated null allele frequencies (Brookfield 2 method) for 13 microsatellite loci from *N. douglasiae* ^[30]^. Bold type indicates significant probability for the presence of null alleles.

| Locus | LZ | DT | PY | GJ | XN | HZ | TH |
| --- | --- | --- | --- | --- | --- | --- | --- |
| Udo1 | 0 | **0.1089** | **0.2266** | **0.1377** | 0.0611 | 0 | 0 |
| Udo2 | 0 | 0 | 0 | 0 | 0 | 0 | 0 |
| Udo3 | 0.0202 | 0 | 0.0345 | 0 | 0 | 0 | 0 |
| Udo4 | 0.1078 | 0.0303 | **0.0934** | 0.026 | 0.0838 | **0.0573** | **0.0573** |
| Udo5 | 0.0681 | 0 | 0 | 0.0892 | 0 | **0.2792** | 0.0684 |
| Udo6 | 0.1078 | 0.0258 | 0 | 0 | 0 | 0 | 0 |
| Udo7 | 0 | **0.1303** | 0.0627 | **0.1084** | 0.0438 | 0 | 0 |
| Udo8 | 0.1045 | 0.034 | 0.0216 | **0.0891** | 0 | 0 | 0 |
| Udo9 | 0 | **0.1184** | 0.0204 | 0.0314 | 0 | 0 | 0 |
| Udo10 | 0.0545 | **0.1475** | **0.1199** | 0.0502 | 0.0765 | **0.2257** | 0.0291 |
| Udo11 | 0.0937 | 0.0706 | 0 | 0.0188 | 0 | 0.0044 | 0.023 |
| Udo14 | 0.0769 | **0.203** | **0.1812** | **0.2021** | **0.2057** | **0.1597** | **0.1911** |
| sst17 | 0 | 0 | 0 | 0 | 0 | 0 | 0 |
