# Supplementary material for: Genetic structure and diversity of Nodularia douglasiae (Bivalvia: Unionida) from the middle and lower Yangtze River drainage
Source: PLoS One. 2017 Dec 20;12(12):e0189737. doi: 10.1371/journal.pone.0189737 (PMC5738091; doi:10.1371/journal.pone.0189737)
Supplement: S4 Table — (DOCX) [file pone.0189737.s006.docx]

**S4 Table.** List of all individual *Nodularia* sp. and outgroups used, collection sites, and GenBank accession codes.

| Species | Country | Code/GenBank |
| --- | --- | --- |
| *Nodularia douglasiae* | Russia | BIV0245/MF975694 |
| *Nodularia douglasiae* | Russia | BIV0244/MF975693 |
| *Nodularia douglasiae* | Russia | BIV1496/MF975696 |
| *Nodularia douglasiae* | Russia | BIV0238/MF975692 |
| *Nodularia douglasiae* | Russia | BIV1491/MF975695 |
| *Nodularia douglasiae* | Russia | BIV1533/ MF975698 |
| *Nodularia douglasiae* | Russia | BIV1532/ MF975697 |
| *Nodularia douglasiae* | Japan | BIV0008/MF975689 |
| *Nodularia douglasiae* | Japan | BIV0007/MF975688 |
| *Nodularia douglasiae* | South Korea | GQ451863 |
| *Nodularia douglasiae* | South Korea | GQ451862 |
| *Nodularia sinuolata* Martens, 1905 | South Korea | GQ451864 |
| *Nodularia nipponensis* Martens, 1877 | Japan | BIV0021/MF975691 |
| *Nodularia nuxpersicae* Dunker, 1848 | Vietnam | KX822654 |
| *Unio gibbus* Spengler, 1793 | Morocco | KX822671 |
| *Unio tumidus* Philipsson, 1788 | Ukraine | KX822672 |
| *Unio pictorum* (Linnaeus, 1758) | USA | KC429109 |
| *Unio crassus* Philipsson, 1788 | France | KC703878 |
| *Acuticosta chinensis* (Lea, 1868) | China | KJ434469 |
| *Solenaia oleivora* (Heude, 1877) | China | KJ434516 |
| *Cuneopsis rufescens* (Heude, 1874) | China | KJ434526 |
| *Cuneopsis heudei* (Heude, 1874) | China | KJ434494 |
| *Lanceolaria grayana* (Lea, 1834) | China | KJ434525 |
| *Schistodesmus lampreyanus*(Baird & Adams, 1867) | China | KJ434510 |
| *Anemina arcaeformis* (Heude, 1877) | China | KJ434479 |
